# Methylation of CYP1A1 and VKORC1 promoter associated with stable dosage of warfarin in Chinese patients

**DOI:** 10.7717/peerj.11549

**Published:** 2021-06-22

**Authors:** Shiwei He, Yuan Wu, Shuidi Yan, Jumei Liu, Li Zhao, Huabin Xie, Shengxiang Ge, Huiming Ye

**Affiliations:** 1 Department of Clinical Laboratory, Women and Children’s Hospital, School of Medicine, Xiamen University, Xiamen, China; 2 National Institute of Diagnostics and Vaccine Development in Infectious Diseases (Xiamen University), School of Public Health, Xiamen University, Xiamen, China; 3 Xiamen Cardiovascular Hospital, School of Medicine, Xiamen University, Xiamen, China; 4 Department of Clinical Laboratory, Zhongshan Hospital, School of Medicine, Xiamen University, Xiamen, China; 5 School of Medicine, Xiamen University, Xiamen, China

**Keywords:** Warfarin, DNA methylation, CYP1A1, VKORC1, Stable dosage

## Abstract

**Objective:**

To investigate the association between DNA methylation and the stable warfarin dose through genome-wide DNA methylation analysis and pyrosequencing assay.

**Method:**

This study included 161 patients and genome-wide DNA methylation analysis was used to screen potential warfarin dose-associated CpGs through Illumina Infinium HumanMethylation 450 K BeadChip; then, the pyrosequencing assay was used to further validate the association between the stable warfarin dose and alterations in the methylation of the screened CpGs. GenomeStudio Software and R were used to analyze the differentially methylated CpGs.

**Results:**

The methylation levels of CpGs surrounding the xenobiotic response element (XRE) within the CYP1A1 promoter, differed significantly between the different dose groups (P < 0.05), and these CpGs presented a positive correlation (r> 0, P < 0.05) with an increase in the stable dose of warfarin. At the VKORC1 promoter, two CpGs methylation levels were significantly different between the differential dose groups (P < 0.05), and one CpG (Chr16: 31106793) presented a significant negative correlation (r <  0, P <  0.05) among different dose (low, medium, and high) groups.

**Conclusion:**

This is a novel report of the methylation levels of six CpGs surrounding the XRE within the CYP1A1 promoter and one differential CpG at the VKORC1 promoter associated with stable warfarin dosage; these methylation levels might be applied as molecular signatures for warfarin.

## Introduction

Worldwide, warfarin is the most used anticoagulant for thrombolytic therapy and thromboprophylaxis (Wardrop & Keeling, 2008), and is prescribed for the prevention and treatment of diverse indications, including atrial fibrillation, deep vein thrombosis, and pulmonary embolism (January et al., 2014; January et al., 2019; Powers et al., 2018; Ruff et al., 2014). However, warfarin has a narrow therapeutic index. In addition, warfarin therapy is subject to large intra- and interindividual variability in dose–response, and the stable dose of warfarin can vary as much as 10-fold among patients (Gage et al., 2008; Kamali & Wynne, 2010). Warfarin doses outside the optimal therapeutic range can frequently lead to serious adverse events, such as hemorrhagic and thromboembolic events in cases of over- and under-dosage, respectively (Tavares, Marcatto & Santos, 2018). Research to improve the accuracy of warfarin dosage has investigated various genetic, clinical, and patient factors (Tavares, Marcatto & Santos, 2018). However, genetic heterogeneity –taken together with the known clinical and patient factors –does not completely explain interindividual differences of warfarin dose requirements (Verhoef et al., 2014).

Previous studies (Baer-Dubowska, Majchrzak-Celińska & Cichocki, 2011; Gomez & Ingelman-Sundberg, 2009) have suggested epigenetics (DNA methylation, miRNA, histone modification, etc.) as a potential explanation of interindividual differences in the drug-dose requirement. Moreover, there are indications (Heyn & Esteller, 2012; Laird, 2003) that alterations in epigenetic markers, specifically DNA methylation, could be used as biomarkers to explain interindividual differences in drug response. Luo et al. (2018) focused on gene module methylation levels and found that alterations in DNA methylation level are significantly associated with a stable dosage of warfarin. Similarly, Wang et al. (2008) explored the role of DNA methylation at the CpG islands of the VKORC1 promoter and showed DNA methylation neither has much effect on VKORC1 expression, nor do the surrounding polymorphisms alter the DNA methylation pattern in VKORC1. Nonetheless, no research has validated the specific CpGs methylation level associated with the stable warfarin dose.

Drawing inspiration from the research route of epigenome-wide association studies (EWAS) (Michels et al., 2013), we used genome-wide DNA methylation analysis and pyrosequencing assay to investigate the association between the stable warfarin dose and alterations in CpGs methylation. Herein, we present the first report on the association between DNA methylation of CYP1A1 and the VKORC1 promoter and the stable dosage of warfarin in Chinese patients.

## Materials & Methods

### Study subjects and study design

We undertook a two-stage case–control study that enrolled patients who were on stable warfarin treatment from a biobank(Planning and preparation in 2010, collection between 2011 and 2018) of Chinese patients treated at the Zhongshan Hospital, School of Medicine, Xiamen University and the Cardiovascular Hospital, School of Medicine, Xiamen University. The Ethics Committee of the School of Medicine, Xiamen University, China approved this study protocol(IRB approval number: KY2014001), and the study was conducted in accordance with the principles of the Declaration of Helsinki.

Patients on stable warfarin treatment were eligible for inclusion in the study if they fulfilled the following criteria: (1) of Chinese ethnicity; (2) requiring long-term warfarin anticoagulant therapy (target INR 2.0–3.5); (3) were 18 years of age or older; and (4) provided written informed consent. Exclusion criteria were: (1) chronic liver failure; (2) use of other anticoagulant drugs; (3) other drugs known to interact with warfarin (according to the report of Gage et al. (2008), eg. Losartan) with the exception of amiodarone, (4) currently undergoing chemotherapy; and (5) alcoholism.

We enrolled 161 patients during the study period. At the first stage, we included 30 patients as the “Screening cohort” to measure genome-wide DNA methylation and screen for potential warfarin dose-associated CpGs among the three groups (low-dose: 10, medium-dose: 10; high-dose: 10). Additionally, at the second stage, 131 patients were enrolled as the “Validation cohort” in the subsequent pyrosequencing to further study the association between the stable warfarin dose and CpG methylation among the three groups (low-dose: 37; medium-dose: 59; high-dose: 35). The study design and patient disposition is shown in File S1.

### Data collection and patient characteristics

Baseline demographics (age, sex, height, weight, and habits of smoking and alcohol intake) and clinical characteristics (combination use of amiodarone, indications for warfarin treatment, and stable dose of warfarin) of study subjects (Table 1) were obtained from electronic medical records and through a follow-up telephone call.

**Table 1:** Characteristics of patients in the screening cohort.

Demographic characteristics	Total (30)	Low dose (10)	Medium dose (10)	High dose (10)	*P*-values
Age (years), mean ± SD	49.07 ± 9.45	52.70 ± 8.47	50.90 ± 10.29	43.60 ± 7.65	0.093
Sex (M%)	14(46.67)	3(30.00)	5(50.00)	6(60.00)	0.534
Height (cm, mean ± SD)	162.86 ± 7.78	160.60 ± 6.62	164.44 ± 8.47	163.54 ± 8.41	0.348
Weigh (kg, mean ± SD)	62.33 ± 7.94	57.70 ± 6.83	66.16 ± 8.42	63.13 ± 6.68	0.053
Smoking, n (%)	2(6.67)	1(10%)	1(10%)	0 (0.00)	1.000
Drinking, n (%)	2(6.67)	1(10%)	0 (0.00)	1(10%)	1.000
Combination use of amiodarone, n (%)	0 (0.00)	0 (0.00)	0 (0.00)	0 (0.00)	
Warfarin stable dose (mg per day), mean ± SD	3.47 ± 1.99	1.40 ± 0.36	3.02 ± 0.35	5.97 ± 0.74	0.000^*^
Indications, n (%)					0.646
Atrial fibrillation	6(20.00)	3(30.00)	1(10.00)	2(20.00)	
Cardiac Valve Replacement	21(70.00)	7(70.00)	8(80.00)	6(60.00)	
Deep Venous Thrombosis	3(10.00)	0(0.00)	1(10.00)	2(20.00)	

**Notes.**

* Significant association with *P* ≤ 0.05.

Patients with INR in the target range had a minimum interval of 4 weeks between INR measurements. The maximum interval between INR measurements was 12 weeks, and was seen in patients on VKA therapy with consistently stable INRs. Stable warfarin dosage was defined on the basis of three consecutive INR measures in the therapeutic range (2.0–3.5) without any adjustment of warfarin dosing between the minimum and maximum intervals. The mean dose recorded during consecutive visits was defined as the stable dose of warfarin.These definitions were chosen refer to the previous studies (Cai et al., 2017; Tavares et al., 2018).

The study subjects were assigned to “low-dose” (≤2 mg/day), “medium-dose” (2 mg/day to 4 mg/day), and “high-dose” (≥4 mg/day) groups, on the basis of the stable dose of warfarin refer to the previous study in China (Cai et al., 2017). The low dose group and high dose group were defined as case groups,and the medium dose group was defined as control group.

### Sample collection

All patients in this study underwent peripheral blood sampling at the time of study enrollment, which were derived from the remaining samples of medical examination. Blood specimens in commercial vacuum blood collection tubes containing 3.2% sodium citrate were separated by centrifugation at 3500 rpm for 10 min to acquire the peripheral blood cells. After centrifugation, the acquired specimens were stored at 4 degrees freezer. Then, the acquired specimens were transferred to -80 degrees ultra-low temperature freezer in 3 h. The specimens were stored in the ice box during the transfer process. All of the steps(from sampling to freeze) were completed in 6 h.

### Genome-wide DNA methylation analysis

From the screening cohort, on the basis of the stable warfarin dose, three groups (low-dose: 10, medium-dose: 10;high-dose: 10) were matched by the patients, characteristics. We screened for potential warfarin dose-associated CpGs by comparison of genome-wide DNA methylation between the case groups(low dose group and high dose group) and control groups(medium dose group).

We isolated DNA from peripheral blood cell samples by using the QIAamp DNA Blood Mini Kit (Cat.#51306; Qiagen, German). DNA bisulfite conversion was done in a bisulfite batch with the Zymo EZ DNA MethylationTM Kit (Cat.#D5001, Zymo, USA). Genome-wide DNA methylation was measured by Illumina Infinium HumanMethylation 450 K BeadChip (Cat.#WG-314-1001, Illumina, USA).

Data from genome-wide methylation were analyzed using the GenomeStudio Software (version V2011.1, Illumina, San Diego, CA, USA). The average *β*-value was estimated as the proportion of total signal intensity from the methylated-specific probe to represent the methylation level (range 0–1). Delta beta (Δ*β*) was used to represent the difference in methylation levels among the case groups(low dose group and high dose group) and control group(medium dose group). To avoid false positives, the probes were filtered out using a detection *P*-value of less than 0.05. Moreover, probes on the X and Y chromosome were removed to eliminate sex bias. The FDR-adjusted DiffScores were computed to account for multiple testing as well as to limit false positives. The differences in methylation levels among the three dose groups were tested using the Illumina Custom model in GenomeStudio software (Carless, 2015). Significant differences were established with a FDR adjusted DiffScore $\geq {|}13{|}\widetilde {P}\leq 0.05$. The genome-wide methylation analysis was conducted refer to the previous pipelines and workflows (Maksimovic, Phipson & Oshlack, 2017; Morris & Beck, 2015; Morris et al., 2014).

The heatmap of hierarchical clustering for samples and CpGs (FDR adjusted DiffScore $\geq {|}33{|}\widetilde {P}\leq 0.001$) was generated by the Ward method using the R package pheatmap (version 1.0.12). The Volcano plot depicts the genome-wide distribution of hypo- and hypermethylated CpGs based on their Δ *β* and DiffScore using the R package ggplot2 (version 2.2.1). The bar chart represents the distribution of the differential CpGs.

### Gene ontology, pathway, and drug database enrichment analysis

The genes, including differential CpGs, were uploaded to the Web-based gene set analysis toolkit (WebGestalt) to carry out the gene ontology (GO), the Kyoto Encyclopedia of Genes and Genomes (KEGG) pathway database, and the drug database (DrugBank and GLAD4U) enrichment analysis (Liao et al., 2019). The enrichment analysis was conducted based on the parameters(minimum number of IDs in the category: 5; maximum number of IDs in the category: 2000; FDR Method: BH; significance level: Top 10).

To further undertake dimension reduction and screen for the differential CpGs, we used a rapid review (PubMed: “warfarin” AND (“gene” OR “SNP”)) to collect genes that have been shown to encode proteins of the warfarin-associated pharmacokinetics and pharmacodynamics pathways. Then, we obtained the intersection of differential CpGs and warfarin-associated genes, and CpGs within the intersection were chosen as target CpGs for further validation. The gene list is shown in File S1.

### Pyrosequencing assay

To verify some of the significant CpGs from the genome-wide analysis, we selected the target CpGs at the warfarin-associated genes. Thereafter, we used pyrosequencing to detect the methylation levels of CpGs with more samples in the validation cohort (low-dose group: 37; medium-dose group: 59; and high-dose group: 35).

DNA was extracted from peripheral blood cells using the TIANamp Blood DNA Kit (Cat. #DP318, Tiangen, China) and DNA bisulfite conversion was conducted in a bisulfite batch with the EpiTect Bisulfite Kit (Cat. #59104, Qiagen, German); then, the converted DNA used as the template for PCR amplification. All primers for PCR and sequencing were designed by PyroMark Assay Design 2.0 Software (version 2.0.01.15; Qiagen, German). Pyrosequencing was carried out by PyroMark Q24 (Qiagen, German) (Clark et al., 2006; Tost & Gut, 2007). The EpiTect Control DNA and Control DNA Set(Cat. #59695, QIAGEN; German) was used in the pyrosequencing. In addition,the PyroMark Q24 Validation Oligo(Cat. #979203, QIAGEN; German) was also used to check the performance of the PyroMark Q24 system. The protocol of PCR and primers is described in File S1.

### Statistical analysis

Normality of distribution was assessed with the Shapiro–Wilk normality test. The uncertain distribution of data was also considered. Continuous variables were expressed as mean ± standard deviation (SD) or median and interquartile range (IQR) when appropriate. Categorical variables were expressed as numbers and percentages.

The sample size of low dose group and high dose group was less than 50, and the distribution of the continuous variables (eg. CpGs methylation) was uncertain. Moreover, different log transformations (log10, log2, and ln) were used in warfarin stable dose to observe whether this variable transforms to the normality, but this variable was still non-normally distributed. In this case, parametric tests may lead to wrong conclusion. Therefore, the non-parametric tests were used in this study after careful consideration.

Kruskal–Wallis test (for continuous variables) and Fisher’s Exact test (for categorical variables) were used to analyze the differences between the groups (high-, medium-, and low-dose). To adjust for the effect of clinical covariates, including age, sex, height, weight, habits of smoking and alcohol intake, and combination use of amiodarone, to prevent any spurious associations, we used ordinal logistic regression analysis to evaluate the association of every CpG methylation level with the warfarin dose groups using the R package rms (version 6.1.0). Spearman rank correlation analysis was used for the correlation between every CpG methylation level and stable warfarin dose, in differential dose groups, respectively. Using the R package ggplot2 (version 2.2.1), the violin plot and dot plot were combined as a violin–dot plot to represent the differences and distributions of the CpG methylation levels among the three dose groups. The correlation map visualized the results of correlation analysis by using the R package corrplot (version 0.84).

A two-tailed *P*-value less than 0.05 was considered statistically significant in all tests. All statistical analyses (except the genome-wide methylation difference analysis) and plots were conducted using R (Version 3.4.4, R Foundation for Statistical Computing, Vienna, Austria).

## Results

### Patient characteristics

From January 2010 through November 2018, we assigned a total of 161 patients in this study. The baseline demographics and clinical characteristics of the patients included in the screening (*n* = 30) and validation (*n* = 131) cohorts are described in Tables 1 and 2. There were no significant between-group differences in baseline characteristics in the two cohorts.

**Table 2:** Characteristics of patients in the validation cohort.

Demographic characteristics	Total (131)	Low dose (37)	Medium dose (59)	High dose (35)	*P*-values
Age (years), mean ± SD	61.48 ± 9.07	63.14 ± 9.26	62.12 ± 9.00	58.66 ± 8.56	0.078
Sex (M%)	66 (50.4)	19 (51.35)	33 (55.93)	14 (40.00)	0.318
Height (cm, mean ± SD)	163.41 ± 5.65	162.57 ± 6.39	163.23 ± 5.05	164.62 ± 5.76	0.240
Weigh (kg, mean ± SD)	65.61 ± 8.40	64.02 ± 7.35	65.79 ± 8.54	66.96 ± 9.15	0.195
Smoking, n (%)	18 (13.70)	7 (18.92)	8 (13.56)	3 (8.57)	0.420
Drinking, n (%)	11 (8.40)	5 (13.51)	4 (6.78)	2 (5.71)	0.512
Combination use of amiodarone, n (%)	14 (10.70)	6 (16.22)	7 (11.86)	1 (2.86)	0.182
Warfarin stable dose (mg per day, median(IQR))	3.00 (2.00,4.50)	1.50 (1.50,1.88)	3.00 (2.88,3.00)	5.25 (4.50,5.25)	0.000^*^
Indications, n (%):					0.619
Atrial fibrillation	45 (34.35)	14 (37.84)	21 (35.59)	10 (28.57)	
Cardiac valve replacement	36 (27.48)	7 (18.92)	17 (28.81)	12 (34.29)	
Valvular heart disease	32 (24.43)	8 (21.62)	14 (23.73)	10 (28.57)	
Coronary heart disease	14 (10.69)	6 (16.22)	6 (10.17)	2 (5.71)	
Dilated cardiomyopathy	1 (0.76)	1 (2.70)	0 (0.00)	0 (0.00)	
Hypertension	2 (1.53)	0 (0.00)	1 (1.19)	1 (2.86)	
Aortic dissection	1 (0.76)	1 (2.70)	0 (0.00)	0 (0.00)	

**Notes.**

* Significant association with *P* ≤ 0.05.

### Genome-wide DNA methylation analysis

On the array, 473,920 of the 485,577 probes (97.60%) were detected with a detection *P*-value of 0.05 or less, after the probes on the X and Y chromosome were removed. Using differential methylation analysis, we found that 42,549, 26,076, and 3955 CpGs were differentially methylated among the three comparisons (low-dose group−high-dose group; medium-dose group−high-dose group; low-dose group–medium-dose group; FDR-adjusted DiffScore $\geq {|}13{|}\widetilde {P}\leq 0.05$). From the perspective of the distribution of differential CpGs on different gene regions, the differential CpGs among the three comparison pairs are mainly distributed on the Body and IGR regions. In addition, the 1stExon and the 3′ UTR regions are the regions with the least distribution of differential CpGs. From the perspective of differential CpGs on CpG island areas, the differential CpGs among the three comparison pairs are mainly distributed on the opensea area. From the perspective of the distribution of differential CpGs on chromosomes, the differential CpGs among the three comparison pairs are mainly distributed on chromosomes 1 and 6, but less on chromosomes 18 and 21. The detailed distributions of hyper- and hypomethylated CpGs among all comparisons are shown in File S2.

**Figure 1:**
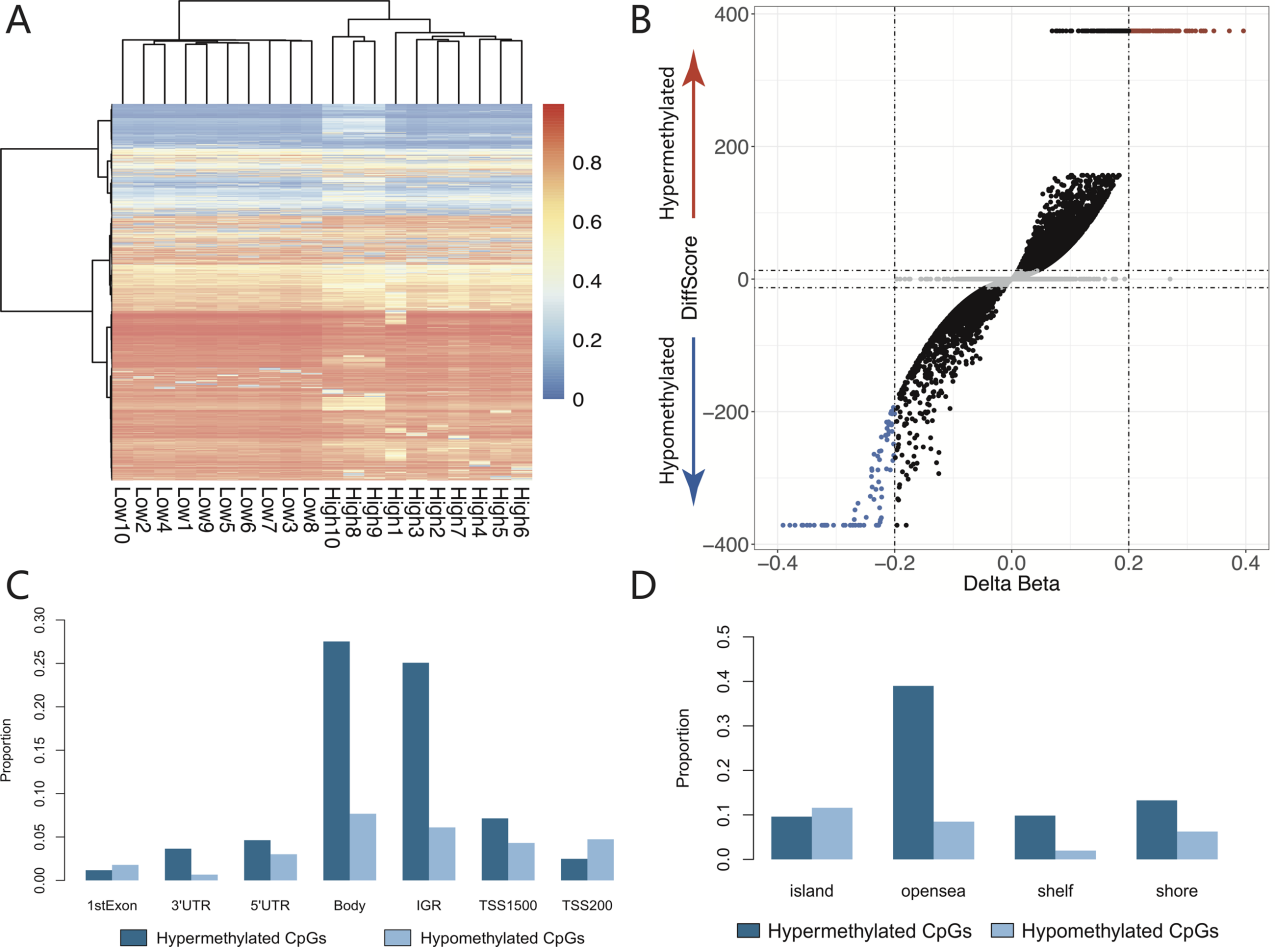
The distributions of differential cpgs between low and high dose group. (A) The chosen differential CpGs (FDR adjusted DiffScore ≥|33| ∼ *P* ≤ 0.001) could better differentiate among each comparison using a hierarchical cluster approach. (B) The genome-wide distribution of hypomethylated and hypermethylated CpGs. (C) As with the distribution of genomic region, compared low dose group with high dose group, 7950 differential CpGs which contained 4098 hypermethylated CpGs and 3852 hypomethylated CpGs were enriched in the promoter region (including TSS1550 and TSS200). (D) Most of differential hypermethylated CpGs (54.38%, 16588/30506) were located in the open sea region, however most of differential hypomethylated CpGs (40.96%,4934/12043) were located in island region.

Furthermore, the selected differential CpGs (FDR-adjusted DiffScore $\geq {|}33{|}\widetilde {P}\leq 0.001$) could better differentiate among each comparison through a hierarchical cluster approach (Fig. 1A). The genome-wide distribution of hypo- and hypermethylated CpGs is shown in Fig. 1B. As with the distribution of the genomic region, when we compared the low-dose group with the high-dose group, 7950 differential CpGs, which contained 4098 hyper- and 3852 hypomethylated CpGs, were enriched in the promoter region (Fig. 1C). Most of differential hypermethylated CpGs (54.38%; 16,588/30,506) were located in the open sea region, whereas most of the differential hypomethylated CpGs (40.96%; 4934/12,043) were located in the island region (Fig. 1D).

### Gene ontology, pathway, and drug database enrichment analysis

To better understand the function of the methylation-altered genes revealed in this study, we conducted enrichment analyses of the GO and KEGG pathway database and the drug database (DrugBank and GLAD4U).

In the GO enrichment analysis, we found 10 biological process terms were significantly enriched (FDR-adjusted *P*-value <0.05) among the 8678 differential genes associated with a warfarin stable dose. All the enrichment analyses are shown in File S3.

To further undertake dimension reduction and screening of the differential CpGs, we undertook a rapid review and verification of the results of genome-wide DNA methylation and chose cg13570656, cg12101586, and cg22549041 –three of the significantly differential CpGs at the CYP1A1 (Cytochrome P450, Family 1, Member A1) gene and cg04263740 at the VKORC1L1 (vitamin K epoxide reductase complex, subunit 1-like 1) gene as the target CpGs. CYP1A1 has been investigated to ascertain its role in R-warfarin metabolism (Zhang et al., 1995). Moreover, human VKORC1L1 was confirmed to be involved in the vitamin K metabolism system, where it can reduce inactive vitamin K 2,3-epoxide to active vitamin K; this system is the target of warfarin that is widely used in thrombolytic therapy and prophylaxis (Czogalla et al., 2018).

In an exploratory attempt in this validation study, as a post hoc analysis, we considered cg09155044 at the promoter of the VKORC1 (vitamin K epoxide reductase complex subunit 1) gene. There are three reasons why we chosen cg09155044 and the surrounding CpGs in this study, although there is no significant difference between the groups on genome-wide DNA methylation analysis. Firstly, DNA methylation at the promoter has been shown to directly silences gene in previous study (Jones, 2012). Secondly, previous study (Oldenburg, Watzka & Bevans, 2015) have shown that warfarin reduces the available reduced vitamin K and reduces the synthesis of active coagulation factors by inhibiting its target VKORC1. Finally, the cg09155044 and the surrounding CpGs are all located on the promoter of the VKORC1. Based on these considerations, we assume that methylation levels of cg09155044 and the surrounding CpGs may be related to warfarin. To verify our hypothesis, we chosen these CpGs in the validation study as a post hoc analysis. Table 3 presents detailed information on the target CpGs.

### Validation study

To verify some of the significant differentially methylated CpGs from genome-wide analysis, we used pyrosequencing technology to test methylation levels of the target CpGs, from more samples in the validation cohort. In addition, the surrounding CpGs (CA-CB, CF-CG, VA, and VC-VE) were taken into the assay range of pyrosequencing (Table 4 presents detailed information on CpGs).

**Table 3:** The detailed information about the target CpGs.

IlmnID	Gene	Region	Average *β*-value			Low-High		Medium-High		Low-Medium	
			Low	Medium	High	Δ*β*	Diff Score	Δ*β*	Diff Score	Δ*β*	Diff Score
cg13570656	*CYP1A1*	Promotor	0.43	0.34	0.39	0.04	10.50	−0.05	−15.35^*^	0.09	19.66^*^
cg12101586	*CYP1A1*	Promotor	0.47	0.38	0.43	0.04	10.01	−0.05	−18.54^*^	0.09	23.09^*^
cg22549041	*CYP1A1*	Promotor	0.41	0.31	0.34	0.07	28.21^*^	−0.02	−5.55	0.09	25.55^*^
cg04263740	*VKORC1L1*	Body	0.57	0.43	0.46	0.11	62.21^*^	−0.03	−5.68	0.14	65.85^*^
cg09155044	*VKORC1*	Promotor	0.61	0.62	0.62	−0.01	−1.60	0.01	0.47	−0.01	0.01

**Notes.**

IlmnID: the ID of CpG for Illumina Infinium HumanMethylation 450 K BeadChip.

* Significant association with a Diff Score ≥|13| ∼ *P* ≤ 0.05.

Table 4 shows results of the Kruskal–Wallis test and ordinal logistic regression analysis. The DNA methylation levels of CpGs (CA-CC, CE, and CG) were significantly different between the differential dose groups (*P* < 0.05); methylation levels of these CpGs at the CYP1A1 gene increased with an increase in the stable warfarin dose.

After adjusting for clinical covariates, there were significant between-group differences among the three dose groups for several CpGs (CA, CB, VD and VE) at the CYP1A1 gene and VKORC1 gene. However, the CpG(VKL) at the VKORC1L1 gene showed no significant difference among the three dose groups in the Kruskal–Wallis test and on logistic regression analysis.

**Figure 2:**
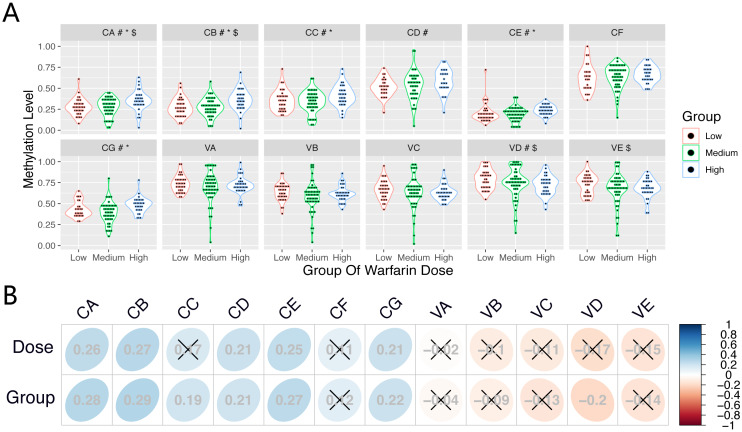
The correlation between CpGs methylation and stable warfarin dose. The correlation between CpGs methylation and stable warfarin dose. (A) The differences and distributions of the CpGs methylation levels among three dose groups. #, *, and $ Significant association with *P* ≤ 0.05 in Spearman Rank Correlation Analysis, Kruskal Wallis Test, and Ordinal Logistic Regression Analysis, respectively; (B) Correlation map showed the correlations between every CpG methylation level and stable warfarin dose, differential dose groups, respectively; × association with *P* ≤ 0.05 in Spearman Rank Correlation Analysis.

Differences of CpG methylation levels (CA-CG and VA-VE) among the three dose groups are shown in Fig. 2A. Table 5 shows the results of Spearman rank correlation analysis. The methylation levels in several CpGs (CA-CB, CD-CE, and CG) at the CYP1A1 gene presented a positive correlation (r > 0, *P* < 0.05) among samples from subjects on the stable warfarin dose. Furthermore, the CpGs (CA-CE, and CG) presented a positive correlation (r > 0, *P* < 0.05) between the methylation levels and different dose groups. At the VKORC1 gene, the methylation levels in VD presented a negative correlation (r < 0, *P* < 0.05) among the different dose groups. However, the CpG (VKL) methylation at VKORC1L1 did not correlate (*P* > 0.05) with the stable warfarin dose variation.The correlation of the CpG (CA-CG and VA-VE) methylation levels among the three dose groups are shown in Fig. 2B.

**Table 4:** The DNA methylation levels and result of difference analysis.

Name of CpG	IlmnID	CHR	MAPINFO	Strand	Low dose median (IQR)	Medium dose median (IQR)	High dose median (IQR)	KW *P*-values	Adjust *P*-values
CA		15	75019185	F	0.27 (0.22, 0.33)	0.29 (0.21, 0.35)	0.35 (0.29, 0.41)	0.003^*^	0.015^**^
CB	cg17852385	15	75019188	F	0.26 (0.19, 0.34)	0.29 (0.21, 0.35)	0.36 (0.29, 0.43)	0.001^*^	0.010^**^
CC	cg13570656	15	75019196	F	0.34 (0.26, 0.42)	0.35 (0.28, 0.42)	0.41 (0.34, 0.49)	0.038^*^	0.141
CD	cg12101586	15	75019203	F	0.52 (0.46, 0.58)	0.56 (0.44, 0.64)	0.57 (0.51, 0.68)	0.108	0.078
CE	cg22549041	15	75019251	F	0.18 (0.15, 0.22)	0.20 (0.14, 0.24)	0.24 (0.20, 0.29)	0.003^*^	0.473
CF	cg11924019	15	75019283	F	0.62 (0.50, 0.70)	0.66 (0.56, 0.73)	0.66 (0.60, 0.74)	0.432	0.575
CG		15	75019288	F	0.40 (0.37, 0.47)	0.40 (0.33, 0.47)	0.49 (0.43, 0.53)	0.002^*^	0.296
VKL	cg04263740	7	65375515	R	0.46 (0.44, 0.87)	0.46 (0.43, 0.87)	0.46 (0.44, 0.88)	0.900	0.989
VA		16	31106779	F	0.73 (0.66, 0.79)	0.70 (0.63, 0.79)	0.71 (0.67, 0.76)	0.635	0.242
VB	cg09155044	16	31106785	F	0.65 (0.58, 0.71)	0.59 (0.54, 0.65)	0.62 (0.57, 0.64)	0.118	0.458
VC	cg01305745	16	31106788	F	0.67 (0.59, 0.73)	0.65 (0.57, 0.71)	0.63 (0.57, 0.67)	0.333	0.351
VD		16	31106793	F	0.80 (0.71, 0.80)	0.76 (0.70, 0.84)	0.74 (0.64, 0.78)	0.090	0.044^**^
VE		16	31106798	F	0.76 (0.63, 0.80)	0.69 (0.63, 0.77)	0.69 (0.64, 0.75)	0.223	0.033^**^

**Notes.**

IlmnID, the ID of CpG for Illumina Infinium HumanMethylation 450 K BeadChip; CHR, the chromosome name; MAPINFO: the site information in chromosome; F, Forward Strand; R, Reverse Strand.

* Significant association with *P* ≤ 0.05 in Kruskal Wallis test.

** Significant association with *P* ≤ 0.05 in ordinal logistic regression analysis.

**Table 5:** Results of spearman rank correlation analysis.

Name of CpG	Stable warfarin dose			Dose group (Low-medium-high)	
	Correlation coefficient	P-Value		Correlation coefficient	*P*-Value
CA	0.258	0.005^*^		0.280	0.002^*^
CB	0.272	0.003^*^		0.292	0.001^*^
CC	0.165	0.079		0.190	0.042^*^
CD	0.212	0.038^*^		0.210	0.039^*^
CE	0.249	0.004^*^		0.266	0.002^*^
CF	0.111	0.247		0.122	0.205
CG	0.213	0.029^*^		0.216	0.027^*^
VKL	0.031	0.730		0.022	0.802
VA	−0.019	0.829		−0.039	0.659
VB	−0.100	0.257		−0.089	0.317
VC	−0.110	0.220		−0.133	0.139
VD	−0.172	0.056		−0.197	0.028^*^
VE	−0.146	0.108		−0.144	0.114

**Notes.**

* Significant association with *P* ≤ 0.05 in spearman rank correlation analysis.

## Discussion

The research route of EWAS provided a reference for our study design, wherein we quantitatively measured genome-wide DNA methylation and screened the potential warfarin dose-associated CpGs among three different dose groups in a small study sample (Michels et al., 2013). Subsequent pyrosequencing further validated the association between the stable warfarin dose and alterations in CpG methylation among differential groups in a larger sample size. Here, we report the methylation levels of six CpGs at the CYP1A1 promoter and one differential CpG at VKORC1 promoter associated with warfarin stable dosage –these might constitute potential molecular signatures for warfarin.

Previous investigations in the field have been of relatively minor scale. Luo et al. (2018) initially showed that alterations in gene module methylation level are significantly associated with the stable warfarin dosage. An earlier investigation of Wang et al. (2008) showed that DNA methylation has little effect on VKORC1 expression. Previously however, no investigation has been undertaken on specific CpG methylation levels that are associated with the stable warfarin dose.

From previous studies (Jones, 2012; Zhang et al., 1995), CYP1A1 contributes to R-warfarin metabolism which is much less potent than S-warfarin, and hypermethylation of the promoter directly silences the gene. Thus, the CYP1A1 methylation may affect the warfarin dose by influencing the expression of CYP1A1 and subsequently affecting the metabolism of R-warfarin. Interestingly, Luo et al. (2017) found that the association between the rs3826041 polymorphism (located upstream of CYP1A1) and warfarin dose. rs3826041 polymorphism may also affect the warfarin dose by influencing the expression of CYP1A1. In this study, the warfarin-associated CpGs within CYP1A1 promoter (chr15:75019185-75019288) are closed to the area of rs3826041(chr15:75018790-75018990), thus, the relation between these CpGs methylation and rs3826041 polymorphism need to be further investigated.

Furthermore, theoretically it can be inferred that the promoter methylation level of CYP1A1 should decline with an increase in the stable warfarin dose. However, the results from our study contradict the theoretical assumptions. In addition, from this result, we can hypothesize that the hypomethylated CpGs within the VKORC1 promoter increased VKORC1 expression, which might have caused a higher need of warfarin dose to maintain therapeutic levels of thrombolytic therapy.

To further explain the contradictory results of the CYP1A1 promoter, we undertook a rapid review of 102 articles from PubMed (“DNA methylation” AND “CYP1A1″, published between 1946 and March 10, 2019) and screened English-only articles related to the methylation of the CYP1A1 gene; duplicates were eliminated through manual review. We found that the CpGs surrounding the xenobiotic response element (XRE) in our study on methylation alterations were associated with exposure to several airborne pollutants (Joubert et al., 2012; Maccani & Maccani, 2015; Shen & Whitlock Jr, 1989; Su et al., 2019), cancers (Anttila et al., 2003; Mitsui et al., 2016), and attention-deficit hyperactivity disorder (Sengupta et al., 2017). It is worth noting that there was no significant differences pertaining to smoking status among the study (low, medium, and high-dose) groups (Tables 1 and 2). In addition, the significantly different CE (chr15:75019251) was the only CpG within the core of XRE (GCGTG).

The critical role of DNA methylation as a biomarker in many diseases has been widely reported. However, one thing is still unclear: the complex interaction between differential DNA methylation and biological phenotype. The researcher’s first task should be to verify whether DNA methylation is the initiating factor or the outcome indicator of biological phenotypic differences? Or whether there are more complex interactions between them. Furthermore, it might be hypothesized that drug tolerance may be caused by alterations in DNA methylation after sustained drug intake, which ultimately leads to alterations in drug dosage through changes in gene expression.

The limitations of the study are as follows: though the patients were matched by the characteristics of patients among differential dose groups, the traditional genetic variables of warfarin were not considered in the study; CYP1A1 contributes to R-warfarin metabolism (R-warfarin is less effective than S-warfarin), then the influence of methylation of CYP1A1 may be limited.

## Conclusions

The methylation levels of surrounding CpGs at the XRE of the CYP1A1 promoter were positively correlated with an increase in the warfarin stable dose. In addition, at the VKORC1 promoter, a CpG (Chr16:31106793) methylation level presented a negative correlation among the different dose (low, medium, and high) groups.

##  Supplemental Information

10.7717/peerj.11549/supp-1 Supplemental Information 1 Supplemental Figure and Tables Detailed information on the study design and methods

10.7717/peerj.11549/supp-2 Supplemental Information 2 The distributions of all comparisons

10.7717/peerj.11549/supp-3 Supplemental Information 3 The results of enrichment analysis

10.7717/peerj.11549/supp-4 Supplemental Information 4 MIAME checklist

## Additional Information and Declarations

### DNA Deposition

The following information was supplied regarding the deposition of DNA sequences: The sequence data is available at figshare: He, Shiwei (2020): Sequence Data. figshare. Dataset. https://doi.org/10.6084/m9.figshare.13490427.v1. The sequence data is the result of pyrosequencing generated from the PyroMark Q24 MDx platform. The raw data (assay sequence) is viewable using PyroMark Q24 MDx Software. Pyrosequencing is an assay method for the DNA methylation at single nucleotide level and single nucleotide polymorphism. The sequence data from pyrosequencing are CpGs or SNPs, they are not eligible to be submitted to GenBank as noncontiguous sequences and sequences with length less than 200 nucleotides.

### Data Availability

The following information was supplied regarding data availability: The data is available at figshare: He, Shiwei (2020): Raw Sequence Data. figshare. Dataset. https://doi.org/10.6084/m9.figshare.13435094
He, Shiwei (2020): Sequence Data. figshare. Dataset. https://doi.org/10.6084/m9.figshare.13490427.v1
He, Shiwei (2020): Raw information of samples in Genome-wide DNA methylation assay.csv. figshare. Dataset. https://doi.org/10.6084/m9.figshare.13370114.v4
He, Shiwei (2020): Raw data of sample methylation in Genome-wide DNA methylation analysis.csv. figshare. Dataset. https://doi.org/10.6084/m9.figshare.13370117.v2.
