# Supplementary material for: Methylation of CYP1A1 and VKORC1 promoter associated with stable dosage of warfarin in Chinese patients
Source: PeerJ. 2021 Jun 22;9:e11549. doi: 10.7717/peerj.11549 (PMC8231338; doi:10.7717/peerj.11549)
Supplement: Supplemental Information 1 — Detailed information on the study design and methods [file peerj-09-11549-s001.docx]

**Supplementary File S1**

*
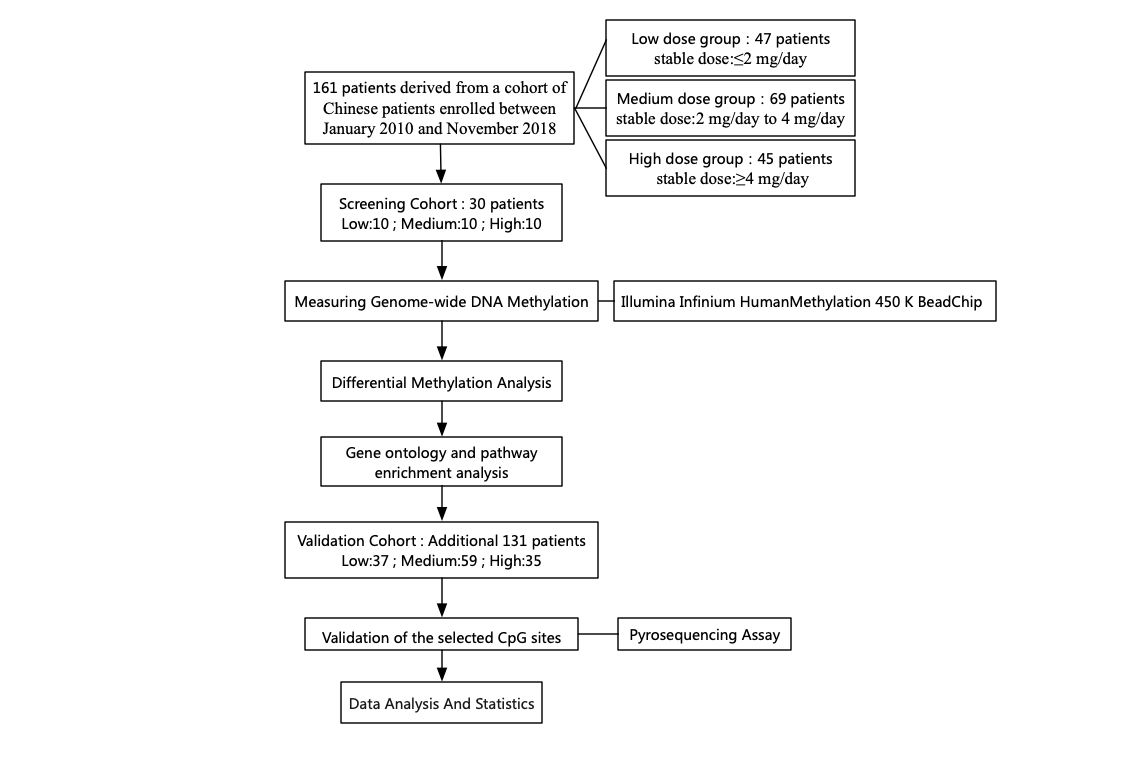
*

**Fig. S1.** The study design of this study

**Table S1.** The primer sequences for PCR and pyrosequencing

| CYP1A1 (first half) | Forward primer | 5’- GTTTTAGTGTTATTTGGTATGGTTTAGT -3’ |
| --- | --- | --- |
| CYP1A1 (first half) | Reverse primer | 5’- ACTATCAACTATATTCCCTTCTCTATC -3’ |
| CYP1A1 (first half) | Pyrosequencing primer | 5’- ATGGTTTAGTTGTTTGTTT -3’ |
| CYP1A1 (second half) | Forward primer | 5’- GGGTTTTTAGGAAAAAAAAAGTTGTAT -3’ |
| CYP1A1 (second half) | Reverse primer | 5’- ACTATCAACTATATTCCCTTCTCTATC -3’ |
| CYP1A1 (second half) | Pyrosequencing primer | 5’- TTTTAGGAAAAAAAAAGTTGTATT -3’ |
| VKORC1 | Forward primer | 5’- AGTGTGTTAGTGTGGTTAATAGTA -3’ |
| VKORC1 | Reverse primer | 5’- CCTCATCTAACCCACAACTTAAA -3’ |
| VKORC1 | Pyrosequencing primer | 5’- GAGATTAGAGGATTAGGG -3’ |
| VKORC1L1 | Forward primer | 5’- TGTGTTATTTAAAGATGATGGTGATGTGA -3’ |
| VKORC1L1 | Reverse primer | 5’- ATTCCAAACCAATTTTATTACCAACATA -3’ |
| VKORC1L1 | Pyrosequencing primer | 5’- ATATAATTAGTTTTGAATTGGGT -3’ |

**Table S2.** Reaction composition using PyroMark PCR Master Mix

| Component | Volume/reaction（μL） | Final concentration |
| --- | --- | --- |
| 2X PyroMark PCR Master Mix | 12.5 |  |
| F1 | 0.5 | 0.2μm |
| R1 | 0.5 | 0.2μm |
| RNase-free water | 8.5 |  |
| Template DNA | 3 | 400～500ng |
| Total volume | 25 | |

**Table S3.** The conditions of PCR

| **Step** | **Temperature** | **Time (minute)** | **Additional comments** |
| --- | --- | --- | --- |
| Initial PCR activation step | 95℃ | 15 | — |
| Denaturation | 94℃ | 0.5 | Number of cycles：45 |
| Annealing | 56℃ | 0.5 |  |
| Extension | 72℃ | 0.5 |  |
| Final extension | 72℃ | 10 | — |

**Table S4** The list of genes related to warfarin

| Number | Gene Symbol | Number | Gene Symbol |
| --- | --- | --- | --- |
| 1 | VKORC1 | 27 | ARSD |
| 2 | CYP2C9 | 28 | PROS1 |
| 3 | CYP4F2 | 29 | ARSF |
| 4 | GGCX | 30 | PRRG2 |
| 5 | CYP2C19 | 31 | F9 |
| 6 | F2 | 32 | STX1B |
| 7 | EPHX1 | 33 | CYP3A5 |
| 8 | CALU | 34 | F11 |
| 9 | ALB | 35 | CYP1A2 |
| 10 | LOC110599569 | 36 | AXL |
| 11 | PROC | 37 | NQO1 |
| 12 | F5 | 38 | CBR1 |
| 13 | VKORC1L1 | 39 | F13A1 |
| 14 | CYP2C18 | 40 | TMEM35B |
| 15 | ORM1 | 41 | SNORA12 |
| 16 | GAS6 | 42 | NR1I2 |
| 17 | F7 | 43 | CYP3A7 |
| 18 | F10 | 44 | CES1 |
| 19 | APOE | 45 | ABCB1 |
| 20 | PROZ | 46 | PRRG3 |
| 21 | CYP3A4 | 47 | PCED1B |
| 22 | ARSE | 48 | METTL7B |
| 23 | MGP | 49 | TMEM134 |
| 24 | UBIAD1 | 50 | TMEM256 |
| 25 | ORM2 | 51 | TMEM242 |
| 26 | CYP2C8 | 52 | SLC31A2 |
|  |  | 53 | CYP1A1 |
