# Supplementary material for: Methylation of CYP1A1 and VKORC1 promoter associated with stable dosage of warfarin in Chinese patients
Source: PeerJ. 2021 Jun 22;9:e11549. doi: 10.7717/peerj.11549 (PMC8231338; doi:10.7717/peerj.11549)
Supplement: Supplemental Information 2 [file peerj-09-11549-s002.pdf]

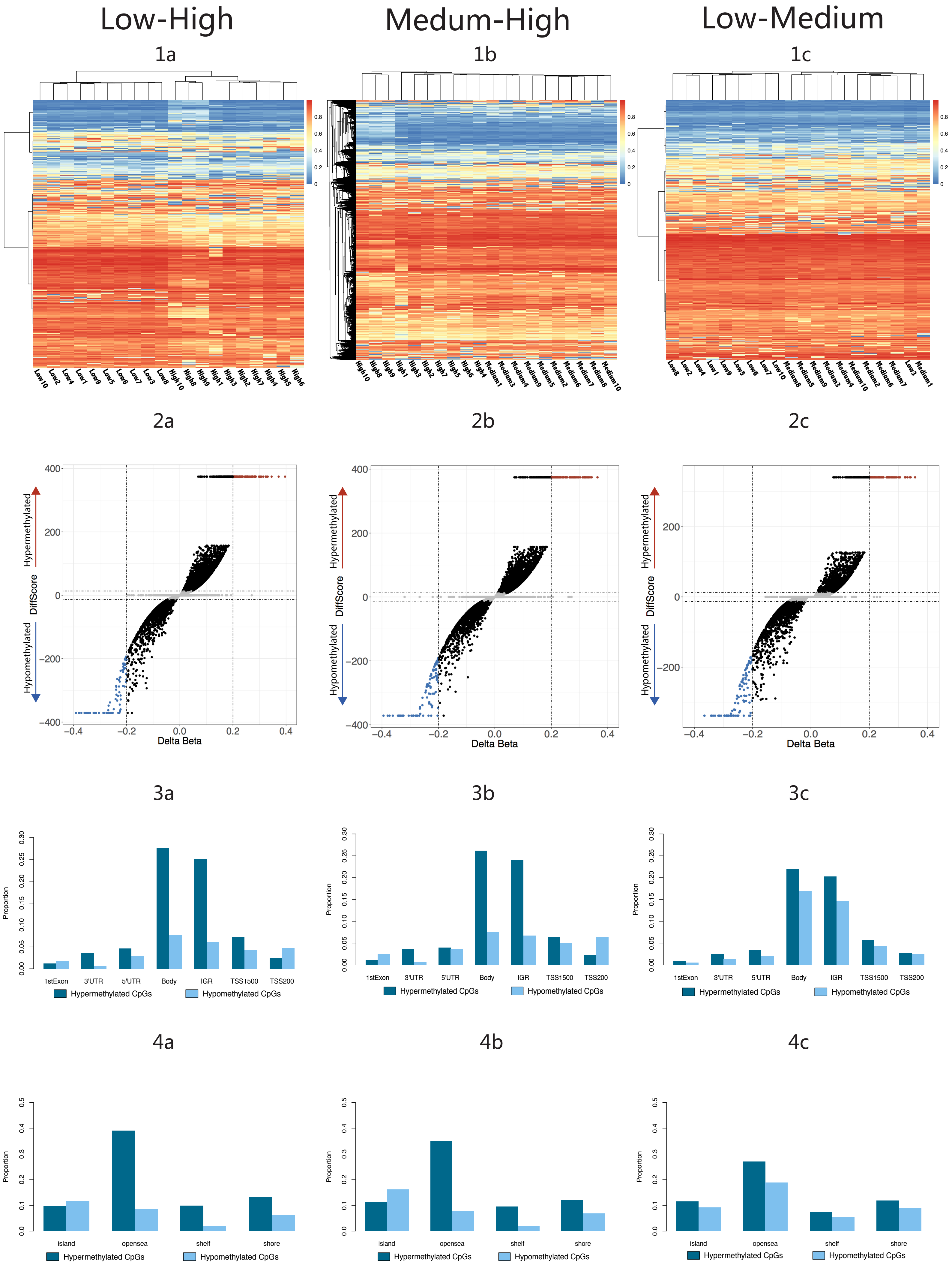

Low-High
Medum-High
Low-Medium
1a
1b
1c
Low10 Low2 Low4 Low1 Low9 Low5 Low6 Low7 Low3 Low8 High10 High8 High9 High1 High3 High2 High7 High4 High5 High6
High10 High8 High9 High1 High3 High2 High7 High5 High6 High4 Medium1 Medium3 Medium4 Medium9 Medium5 Medium2 Medium6 Medium7 Medium8 Medium10
Low8 Low2 Low4 Low1 Low9 Low5 Low6 Low7 Low10 Medium8 Medium5 Medium9 Medium3 Medium4 Medium10 Medium2 Medium6 Medium7 Low3 Medium1
2a
2b
2c
Hypermethylated
DiffScore
Hypomethylated
Delta Beta
3a
3b
3c
Proportion
1stExon
3'UTR
5'UTR
Body
IGR
TSS1500
TSS200
Hypermethylated CpGs
Hypomethylated CpGs
4a
4b
4c
island
opensea
shelf
shore

5a

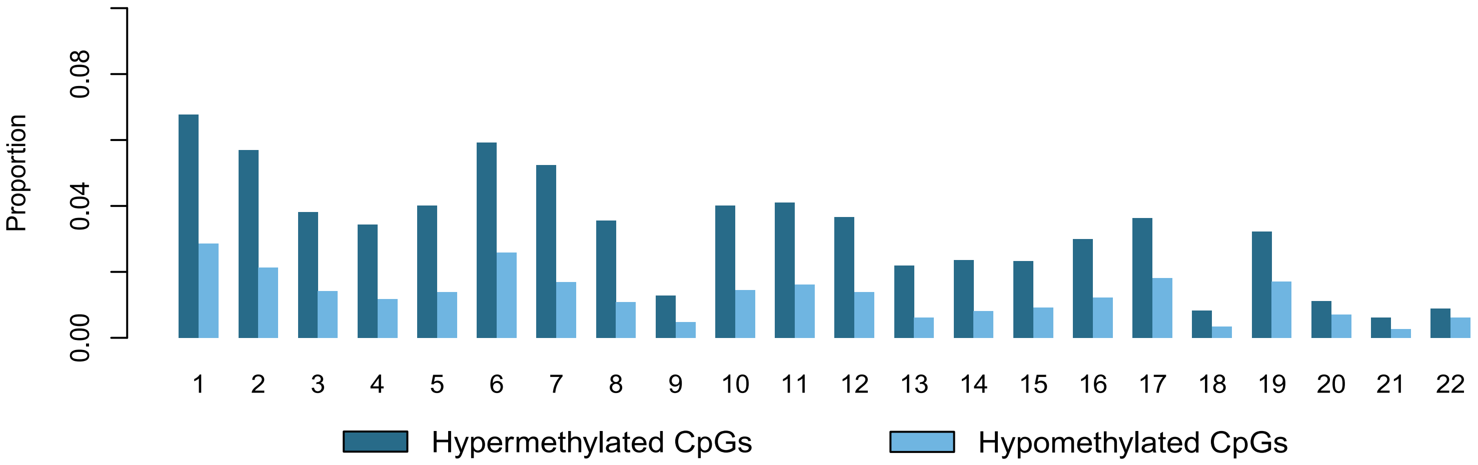
Proportion
0.00
0.04
0.08
1 2 3 4 5 6 7 8 9 10 11 12 13 14 15 16 17 18 19 20 21 22
Hypermethylated CpGs
Hypomethylated CpGs

5b

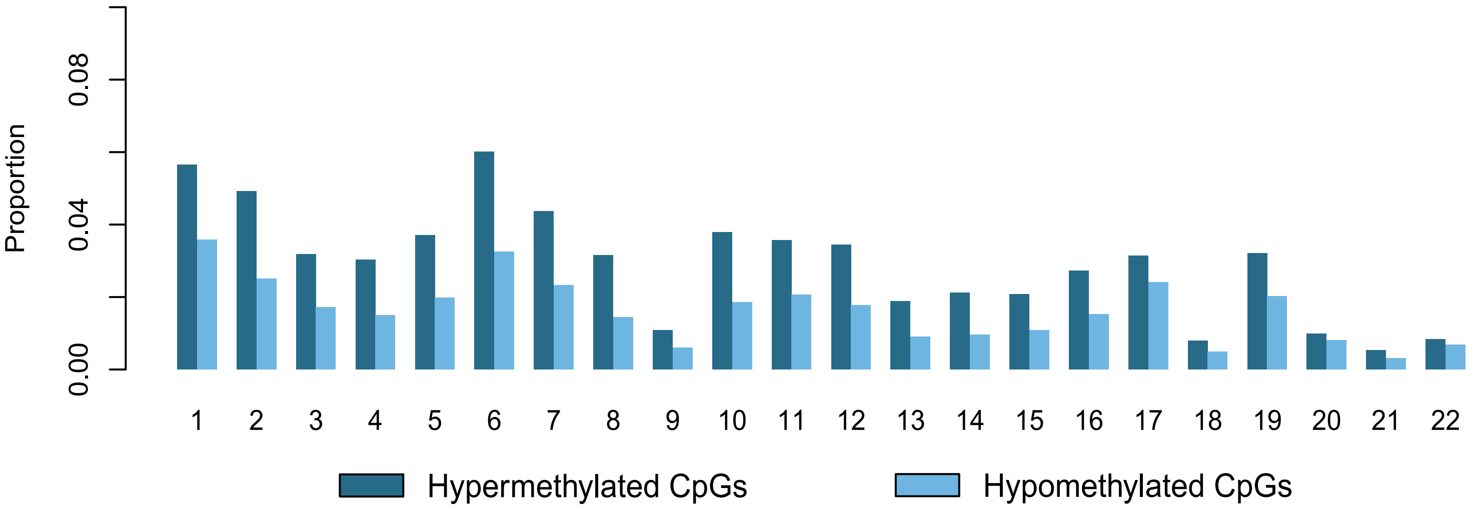
Proportion
0.00
0.04
0.08
1 2 3 4 5 6 7 8 9 10 11 12 13 14 15 16 17 18 19 20 21 22
Hypermethylated CpGs
Hypomethylated CpGs

5c

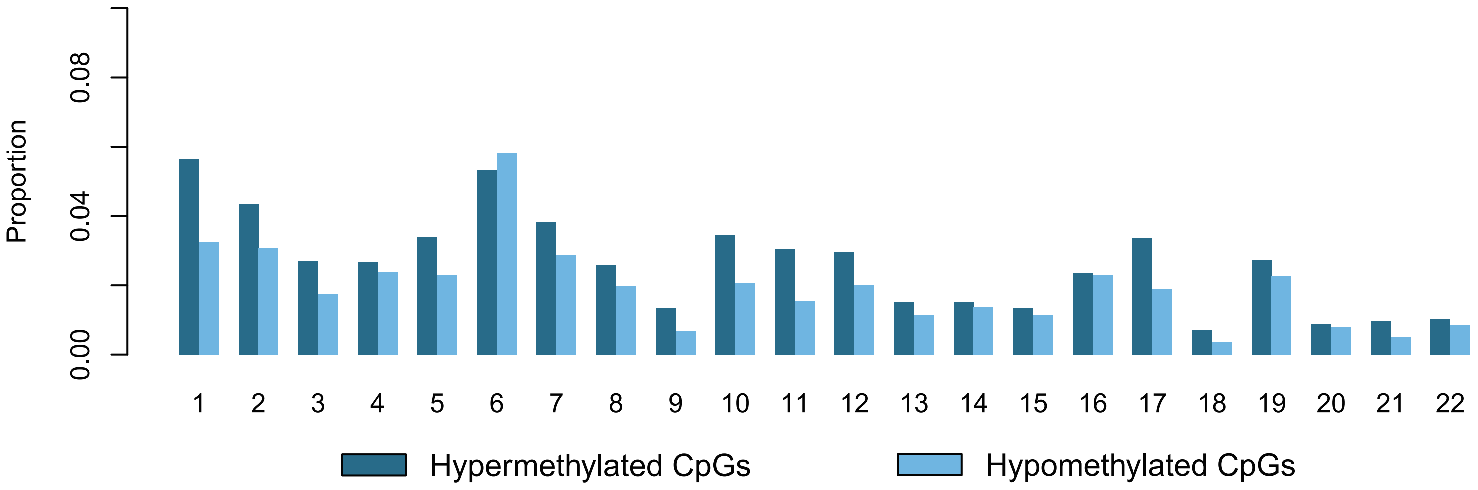
Proportion
0.00
0.04
0.08
1 2 3 4 5 6 7 8 9 10 11 12 13 14 15 16 17 18 19 20 21 22
Hypermethylated CpGs
Hypomethylated CpGs

1a, 1b and 1c: the heatmap of hierarchical clustering for samples and CpGs (Low-High, Medum-High, and Low-Medium).

2a, 2b and 2c: the Volcano plot depicts the genome-wide distribution of hypo- and hypermethylated CpGs based on their Δβ and DiffScore (Low-High, Medum-High, and Low-Medium).

3a, 3b and 3c: the bar chart represents the distribution of the differential CpGs of the genomic region (Low-High, Medum-High, and Low-Medium).

4a, 4b and 4c: the bar chart represents the distribution of the differential CpGs of the CpG island region (Low-High, Medum-High, and Low-Medium).

5a, 5b and 5c: the bar chart represents the distribution of the differential CpGs of the chromosomes (Low-High, Medum-High, and Low-Medium).
