# Supplementary material for: Methylation of CYP1A1 and VKORC1 promoter associated with stable dosage of warfarin in Chinese patients
Source: PeerJ. 2021 Jun 22;9:e11549. doi: 10.7717/peerj.11549 (PMC8231338; doi:10.7717/peerj.11549)
Supplement: Supplemental Information 3 [file peerj-09-11549-s003.docx]

**Supplementary File S3**

The results of enrichment analysis

1 GO analysis

In GO analysis, each biological process, cellular component and molecular function category***(category contains specific terms of enrichment analysis)*** is represented by a red, blue and green bar, repectively. The height of the bar represents the number of IDs in the user list and also in the category.


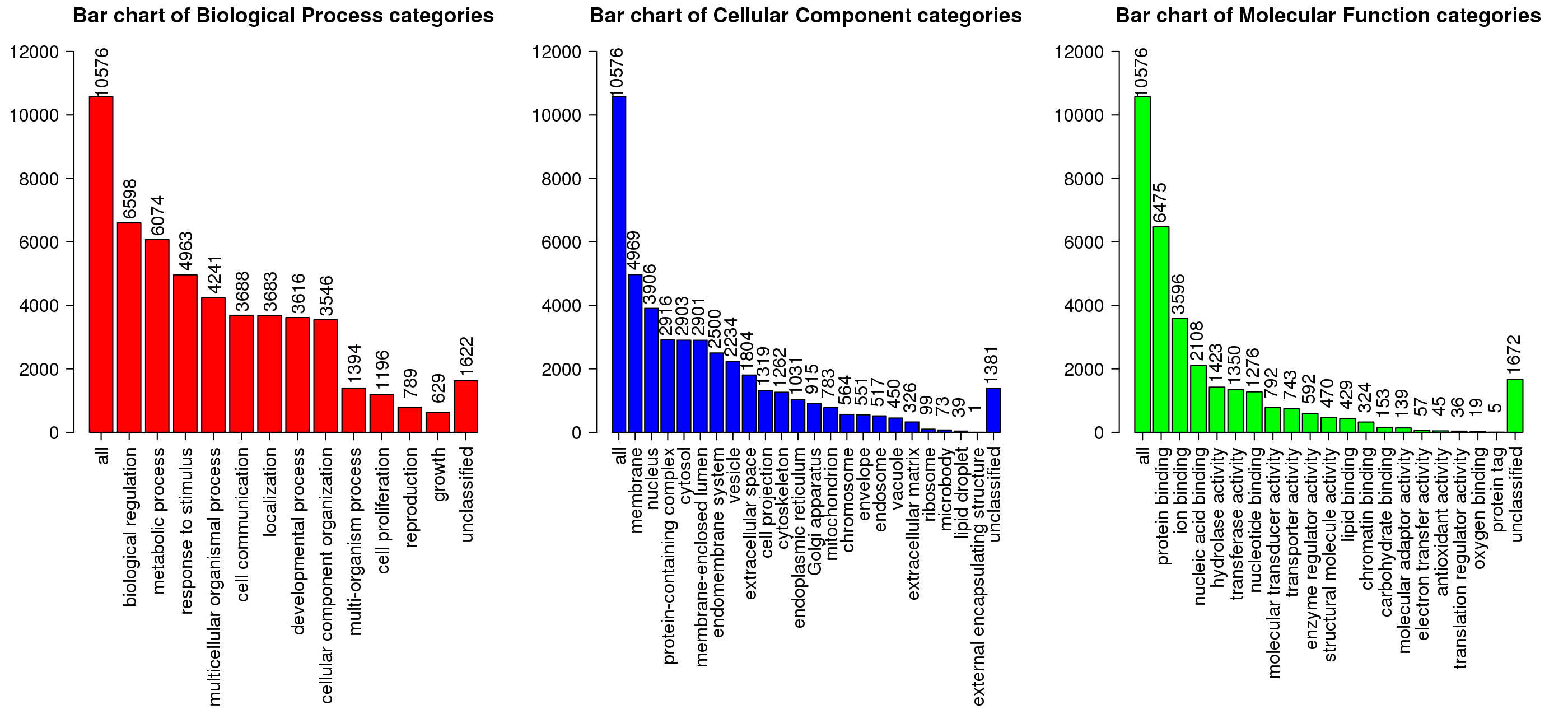
1.1 Terms of GO-Biology Process

1.1.1 Bar chart:10 biological process terms were significantly enriched


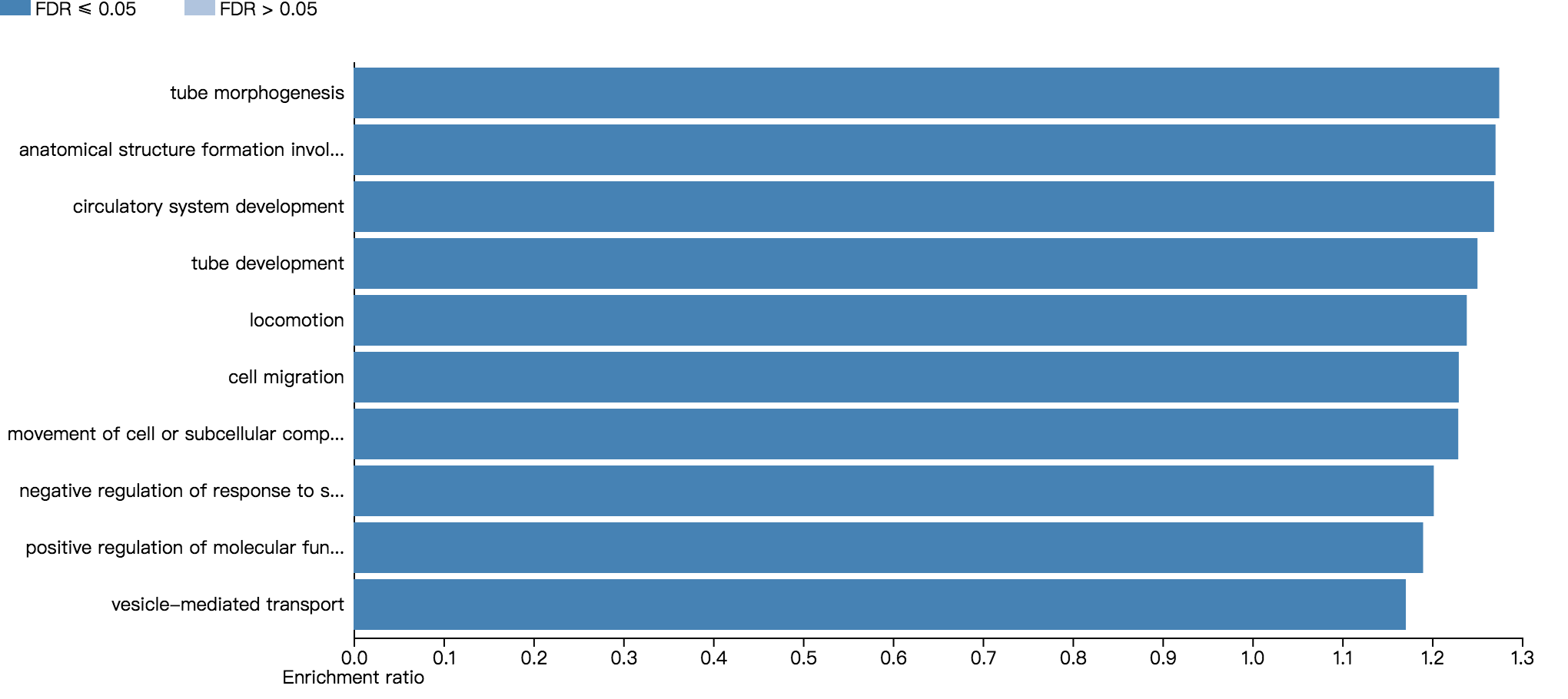


1.1.2 Volcanoplot:10 biological process terms were significantly marked


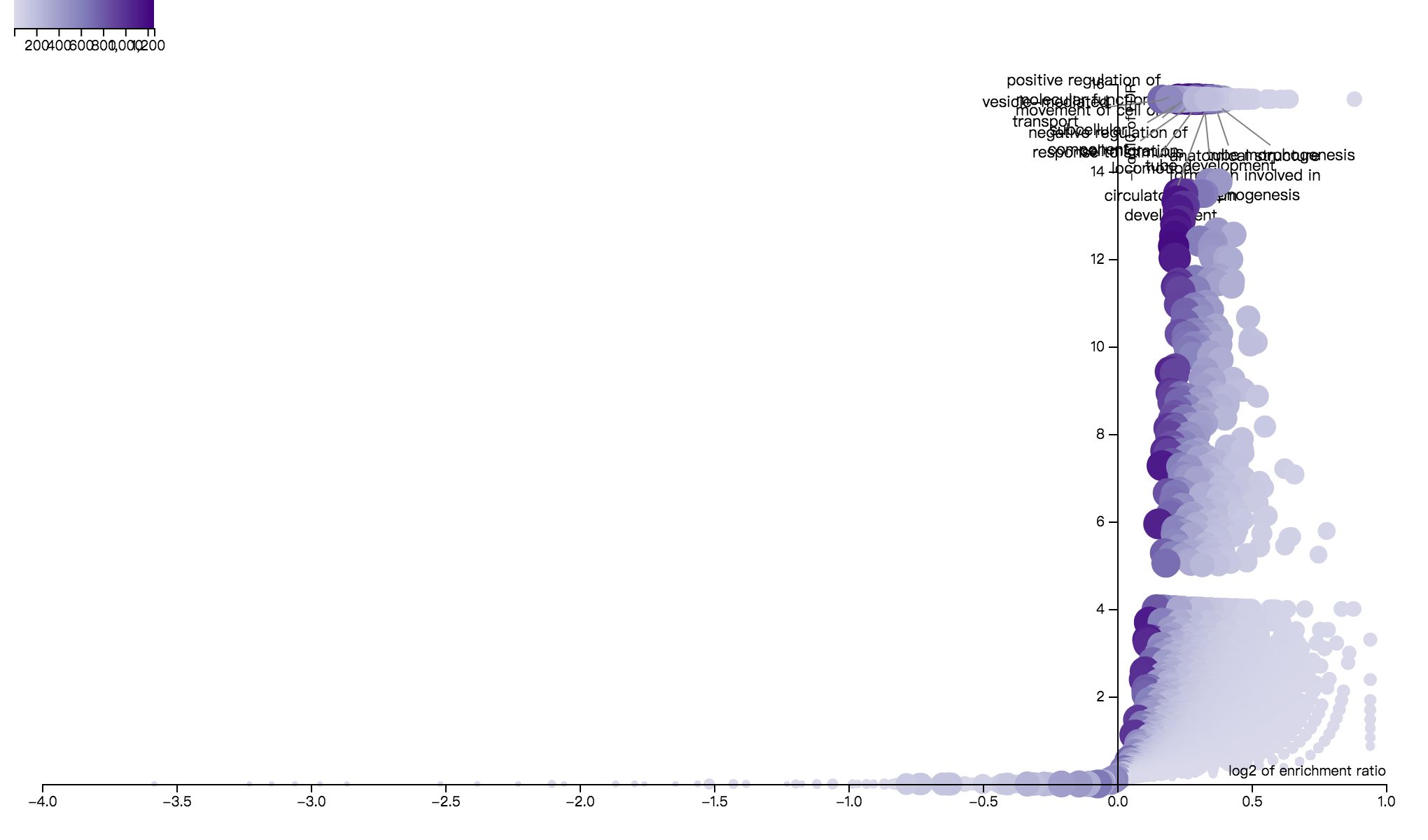


X axis: log2 of enrichment ratio;

Y axis: -log10 of FDR;

Dot shade: overlap value;

Figure captions: 10 terms were significantly marked.

1.1.3 Directed acyclic graph of the GO analysis


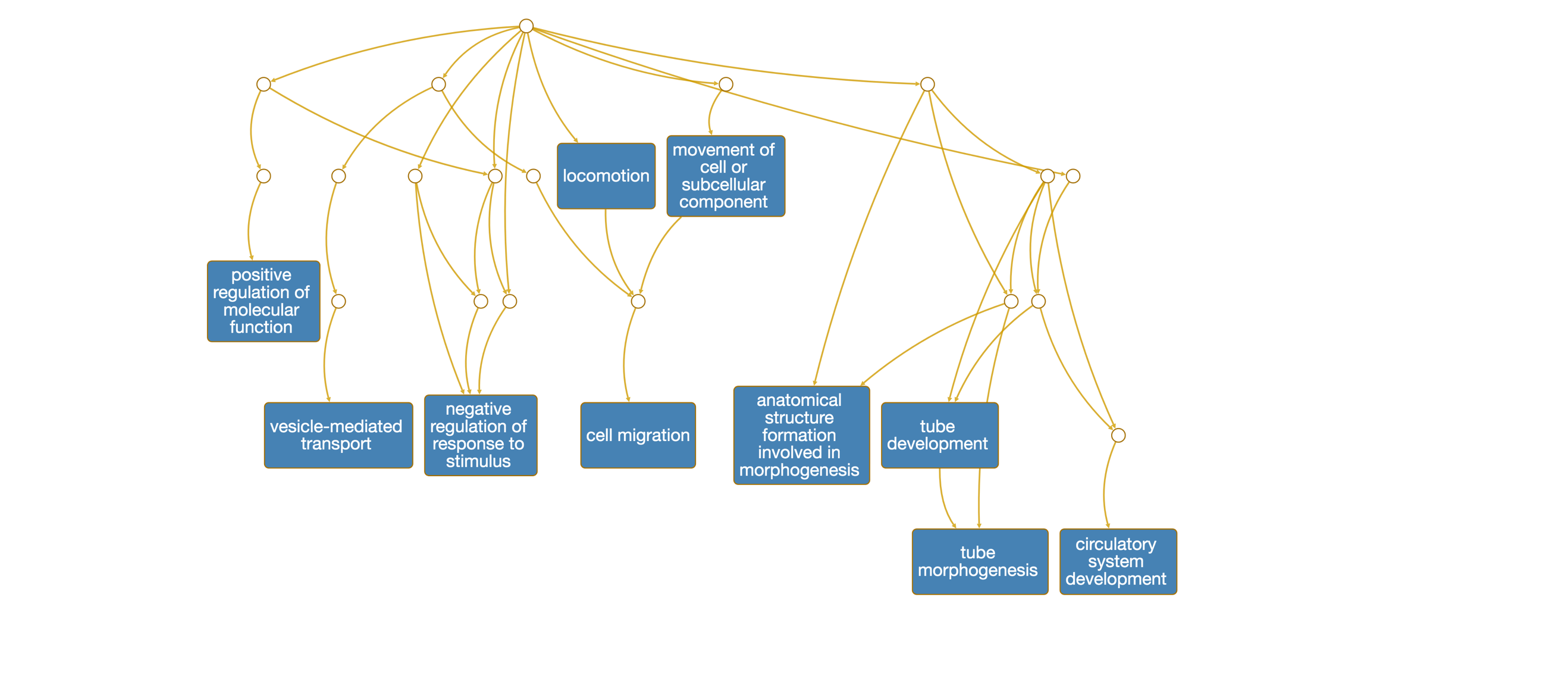


1.2 Terms of GO-Cell Component

1.2.1 Bar chart:10 cell components terms were significantly enriched


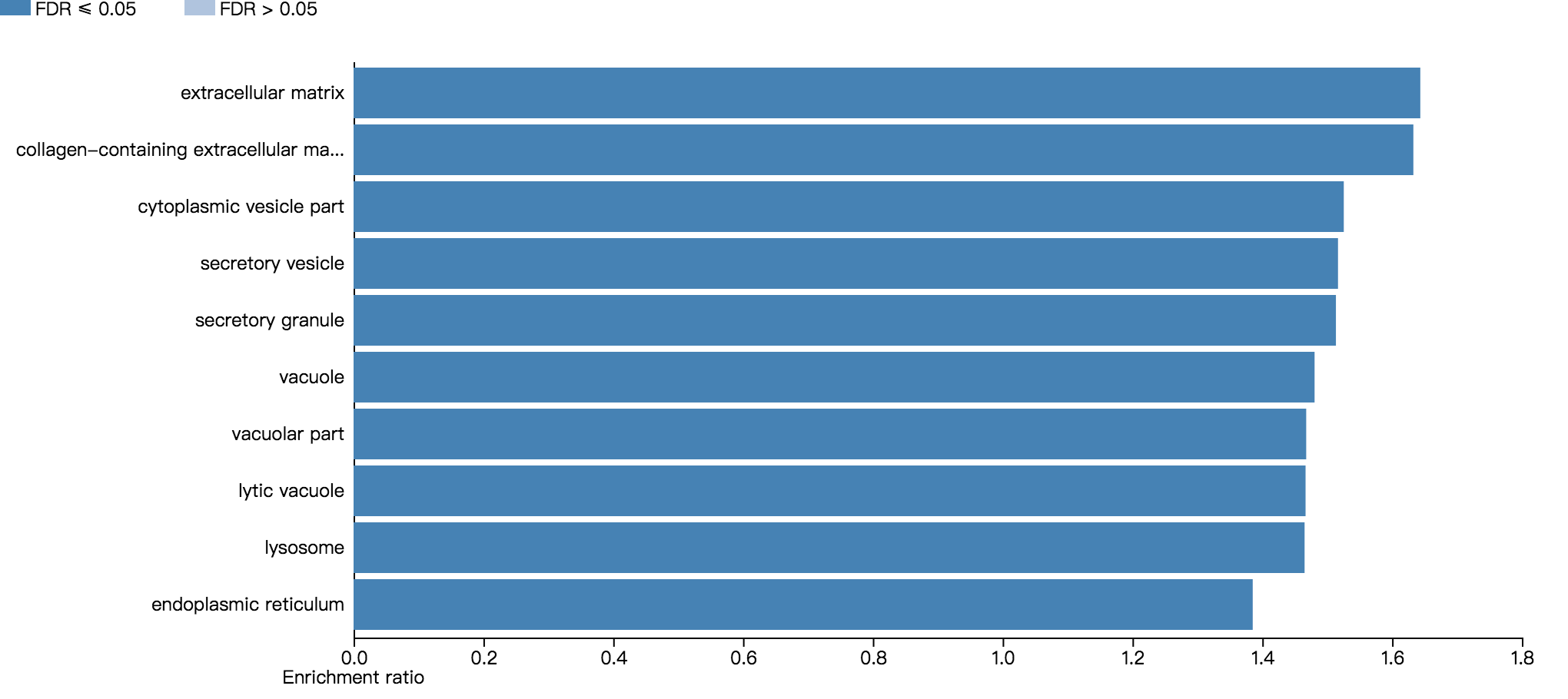


1.2.2 Volcanoplot:10 cell components terms were significantly marked


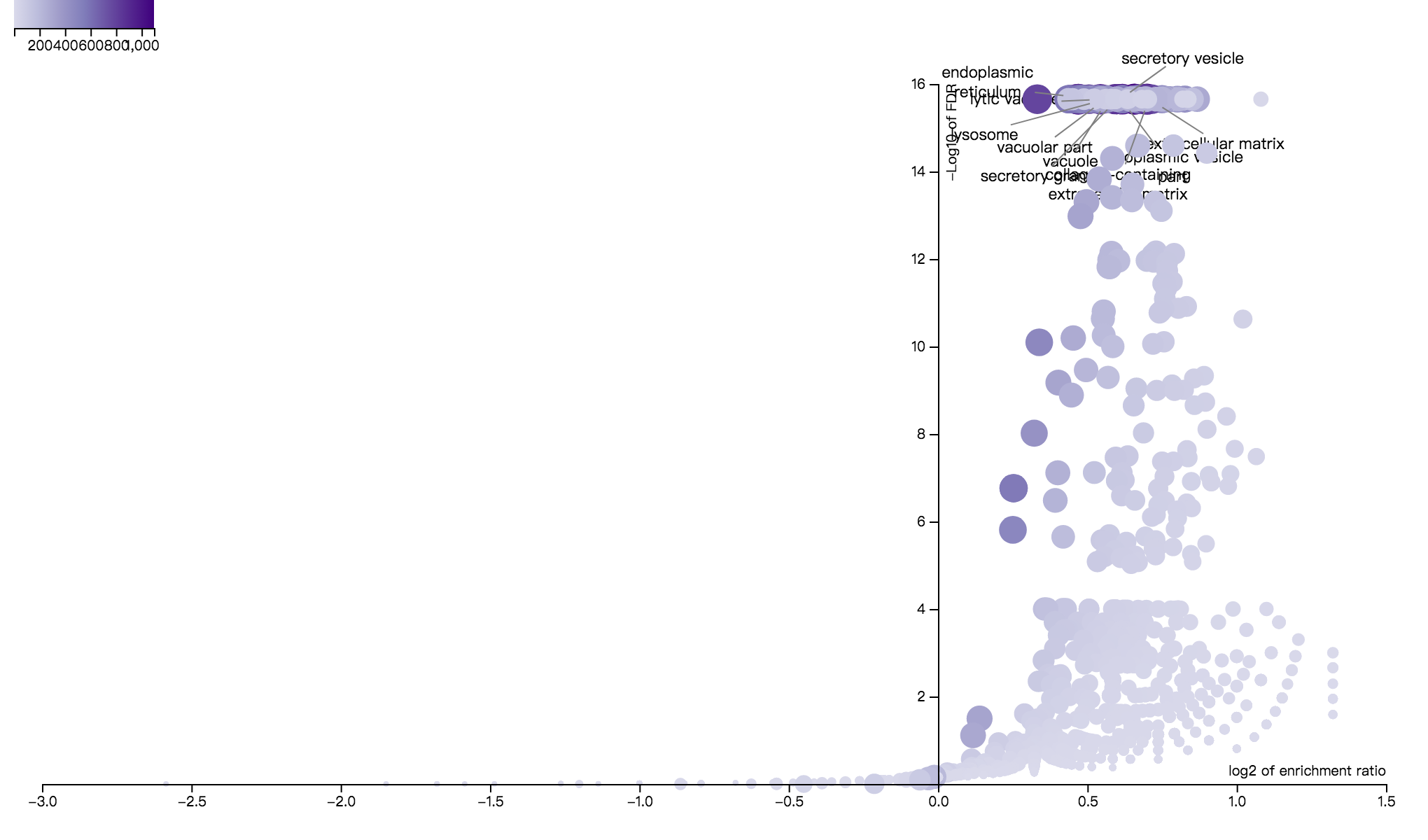
X axis: log2 of enrichment ratio;

Y axis: -log10 of FDR;

Dot shade: overlap value;

Figure captions: 10 terms were significantly marked.

1.2.3 Directed acyclic graph of the GO analysis


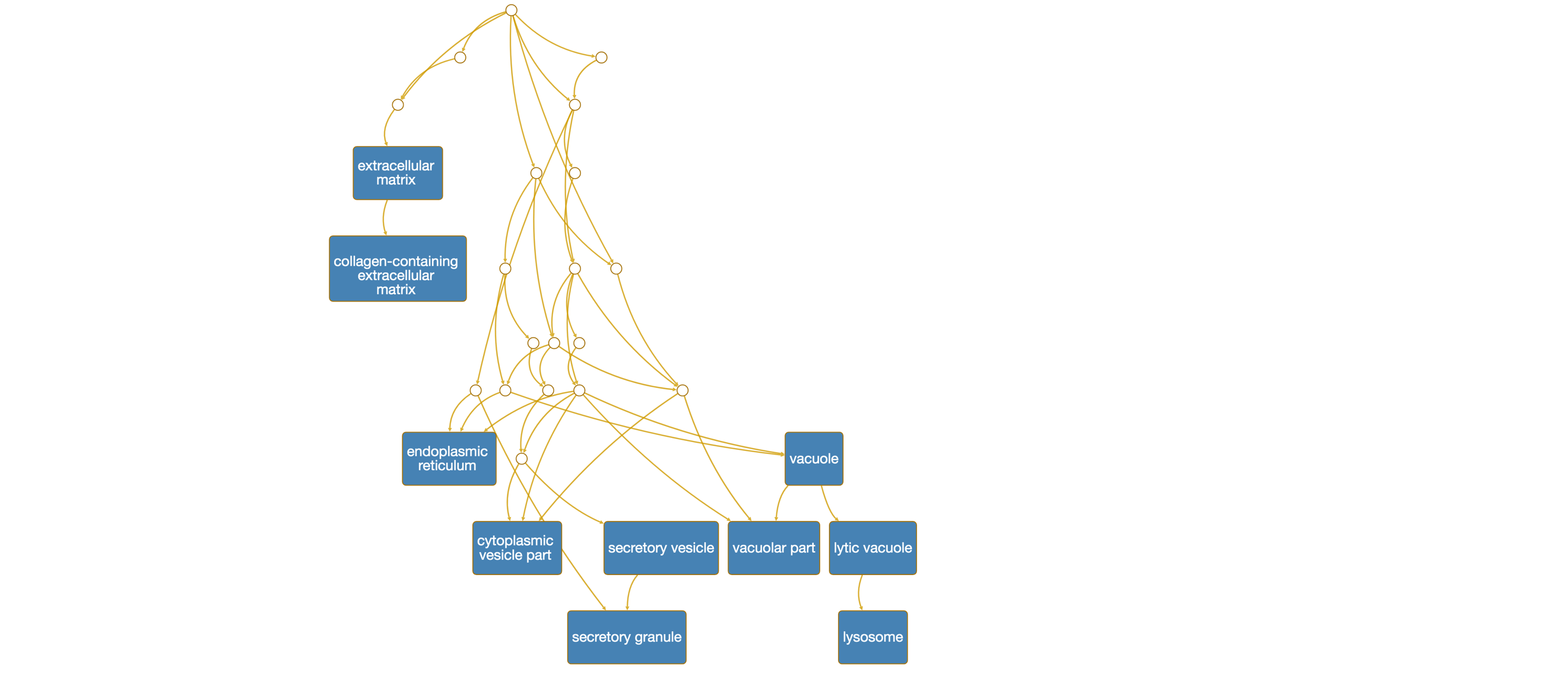


1.3 Terms of GO-molecular function

1.3.1 Bar chart:10 molecular function terms were significantly enriched


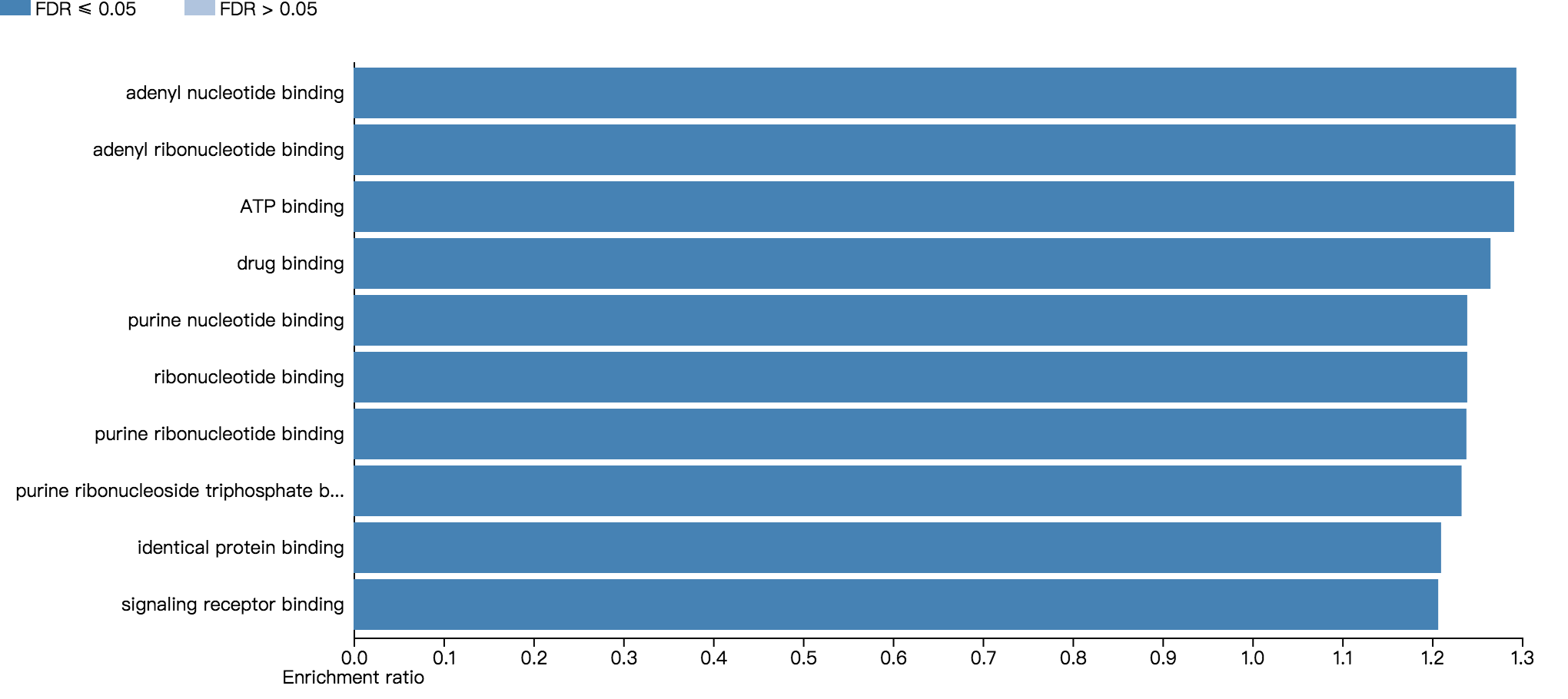


1.3.2 Volcanoplot:10 molecular function terms were significantly marked


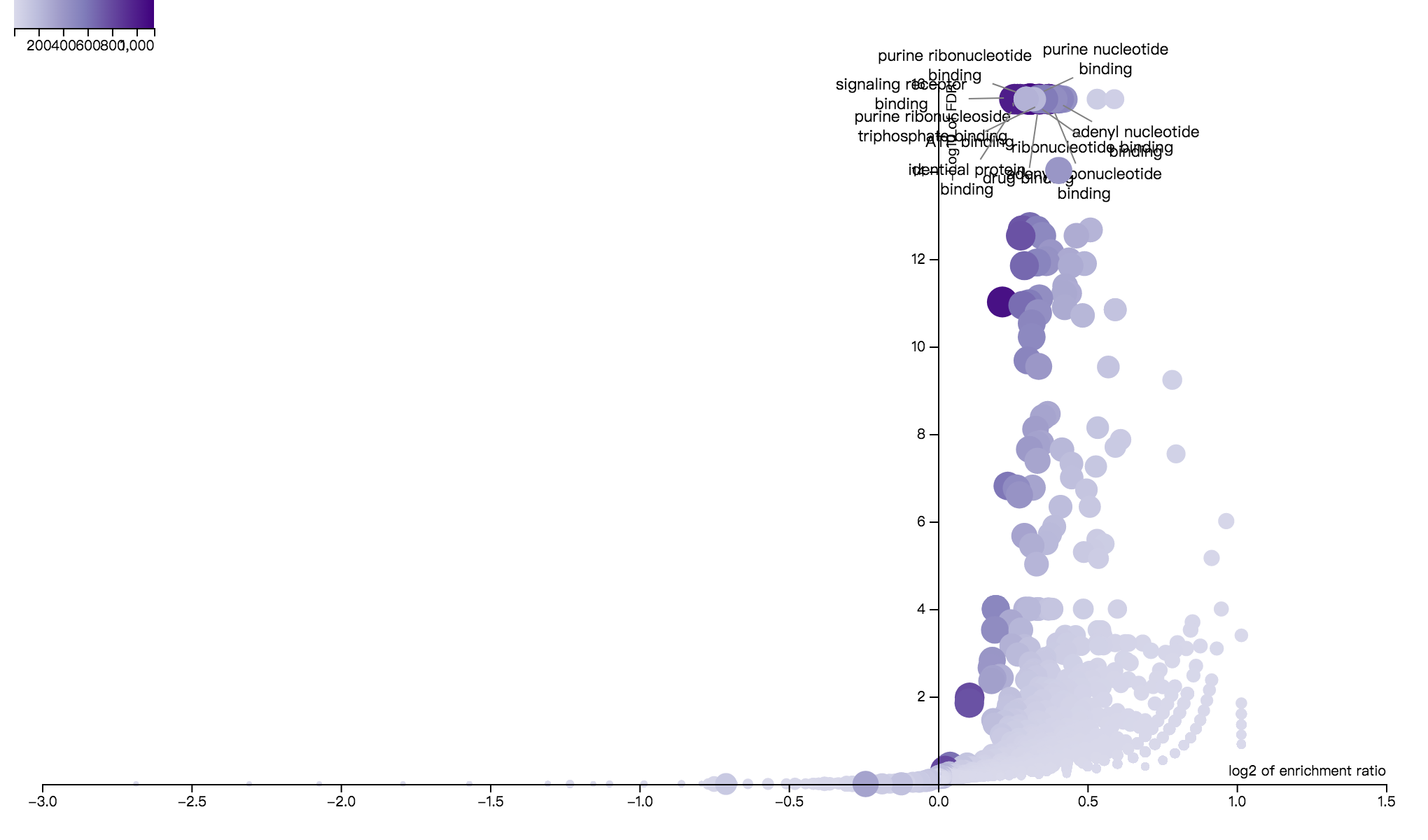


X axis: log2 of enrichment ratio;

Y axis: -log10 of FDR;

Dot shade: overlap value;

Figure captions: 10 terms were significantly marked.


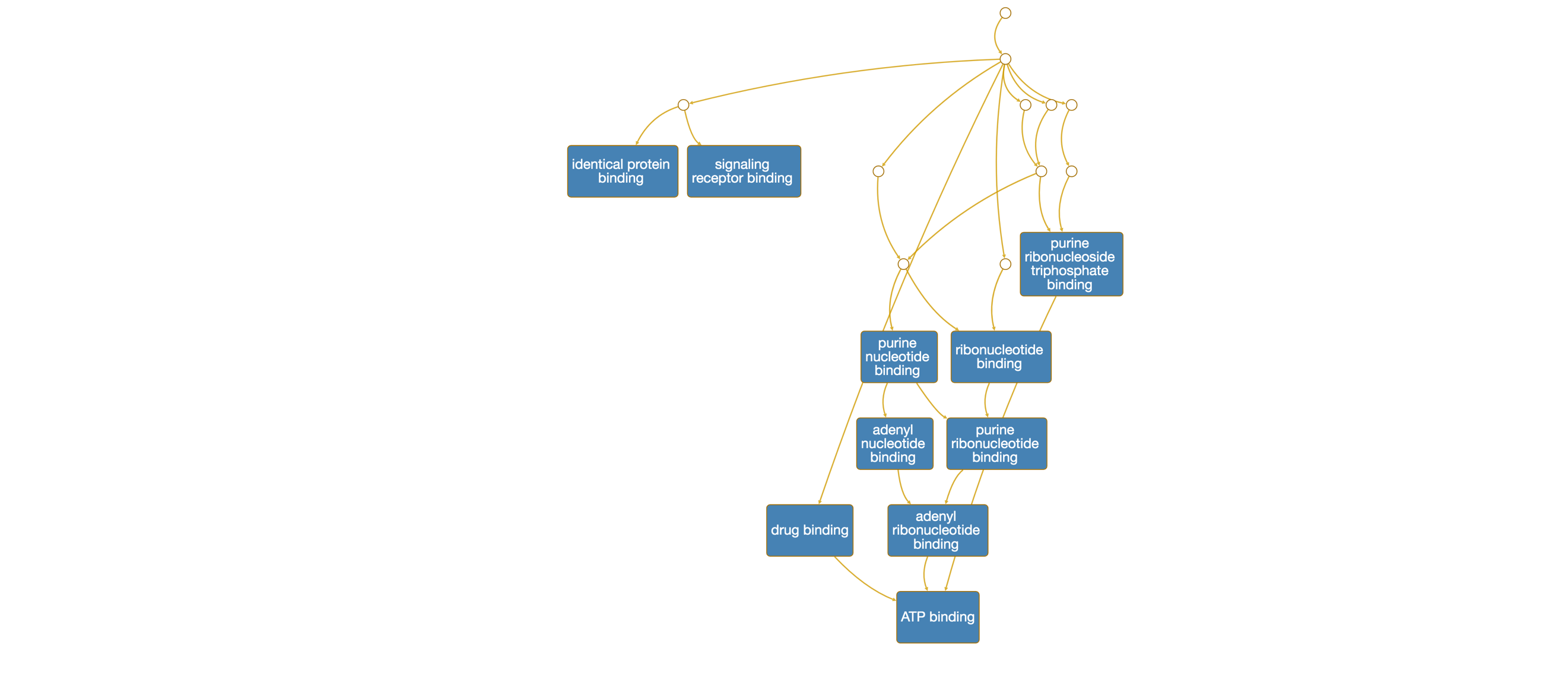
1.3.3 Directed acyclic graph of the GO analysis

2 KEGG

2.1 Bar chart:10 terms were significantly enriched


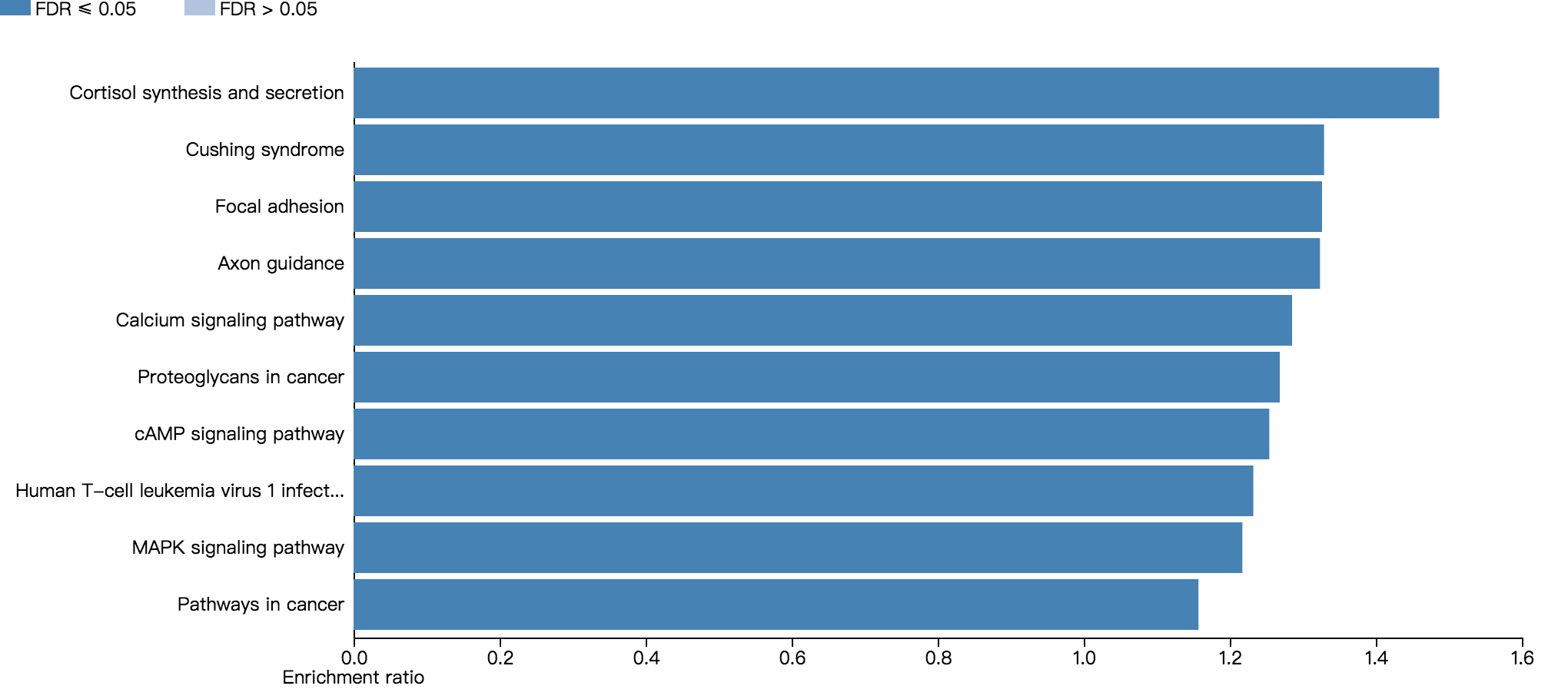


2.2 Volcanoplot:10 terms were significantly marked


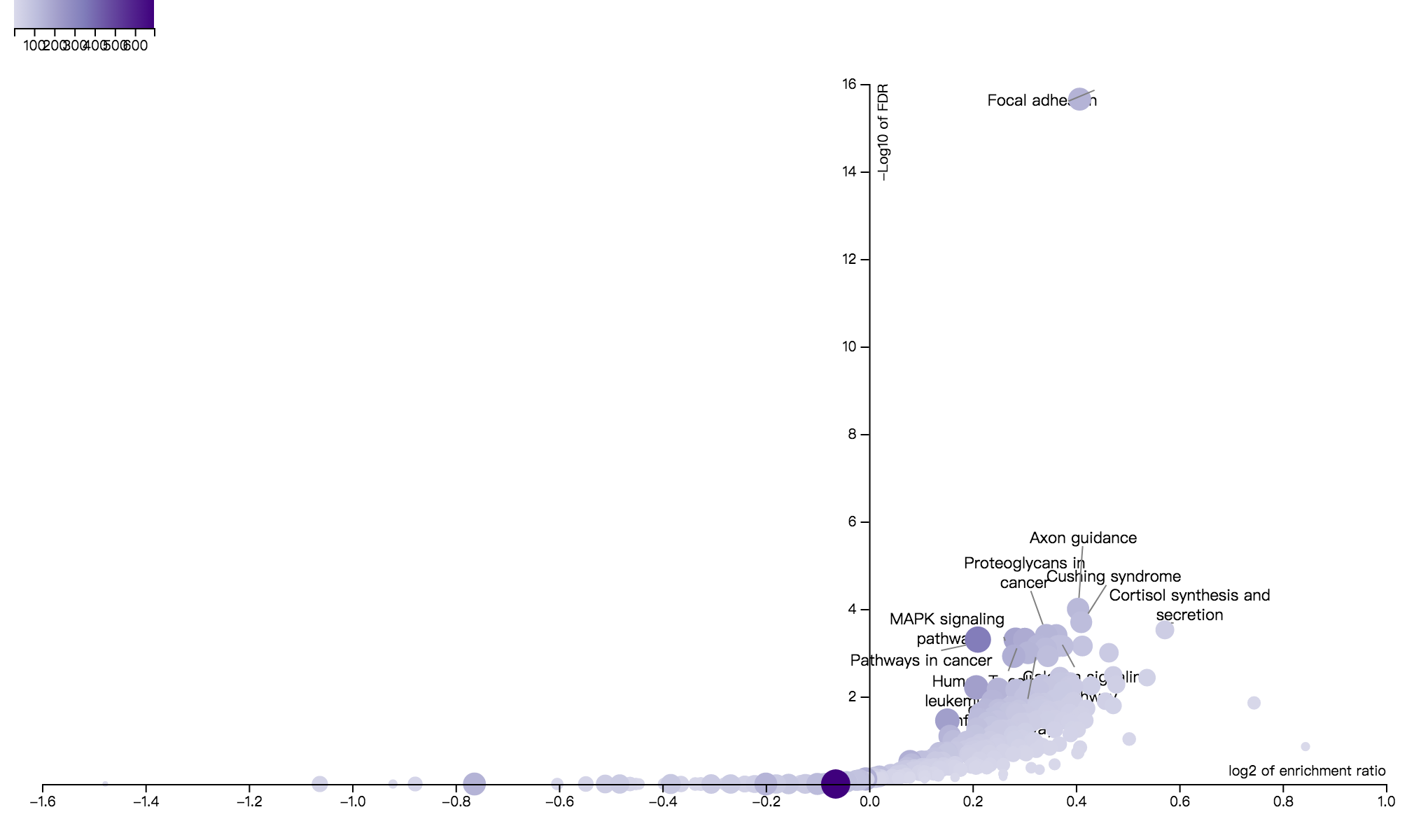


X axis: log2 of enrichment ratio;

Y axis: -log10 of FDR;

Dot shade: overlap value;

Figure captions: 10 terms were significantly marked.

3 Drug Database:Drugbank

3.1 Bar chart:10 terms were significantly enriched


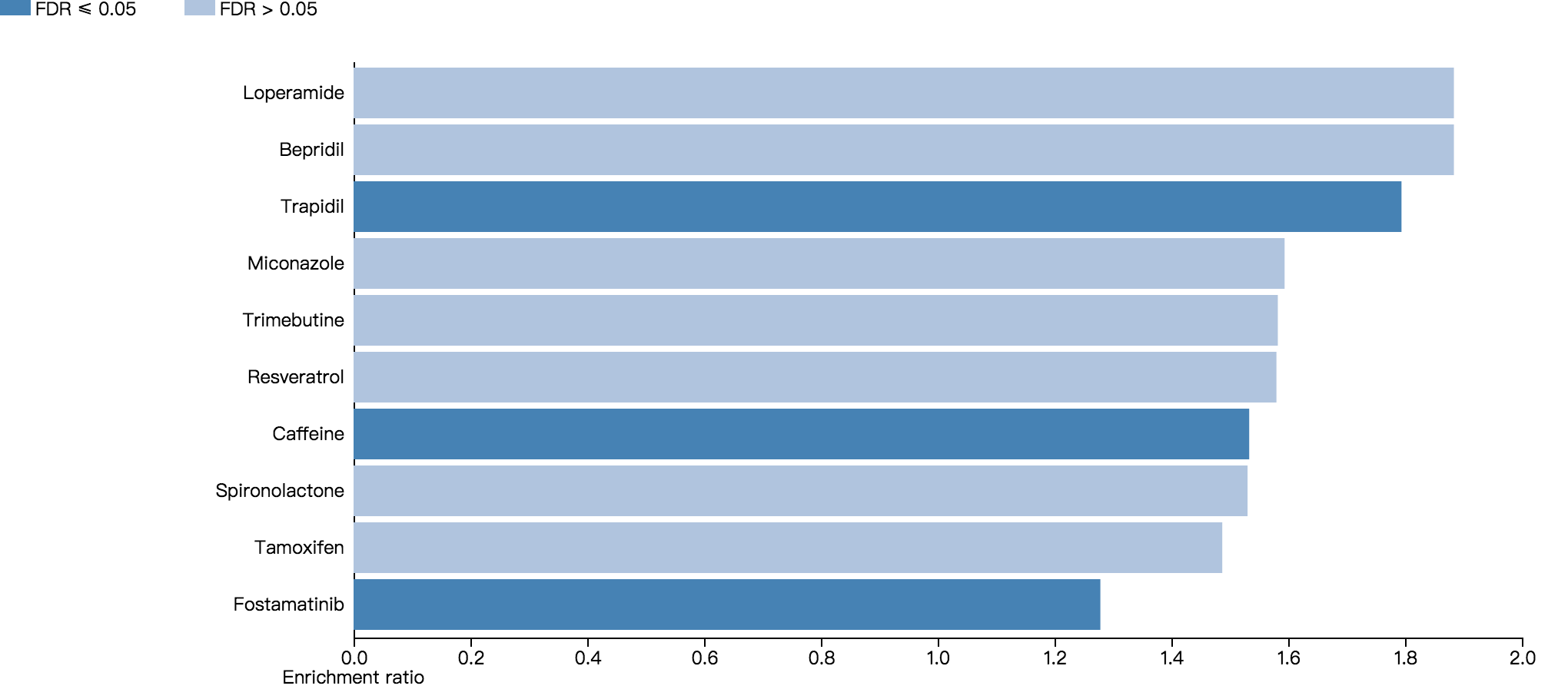


3.2 Volcanoplot:10 terms were significantly marked


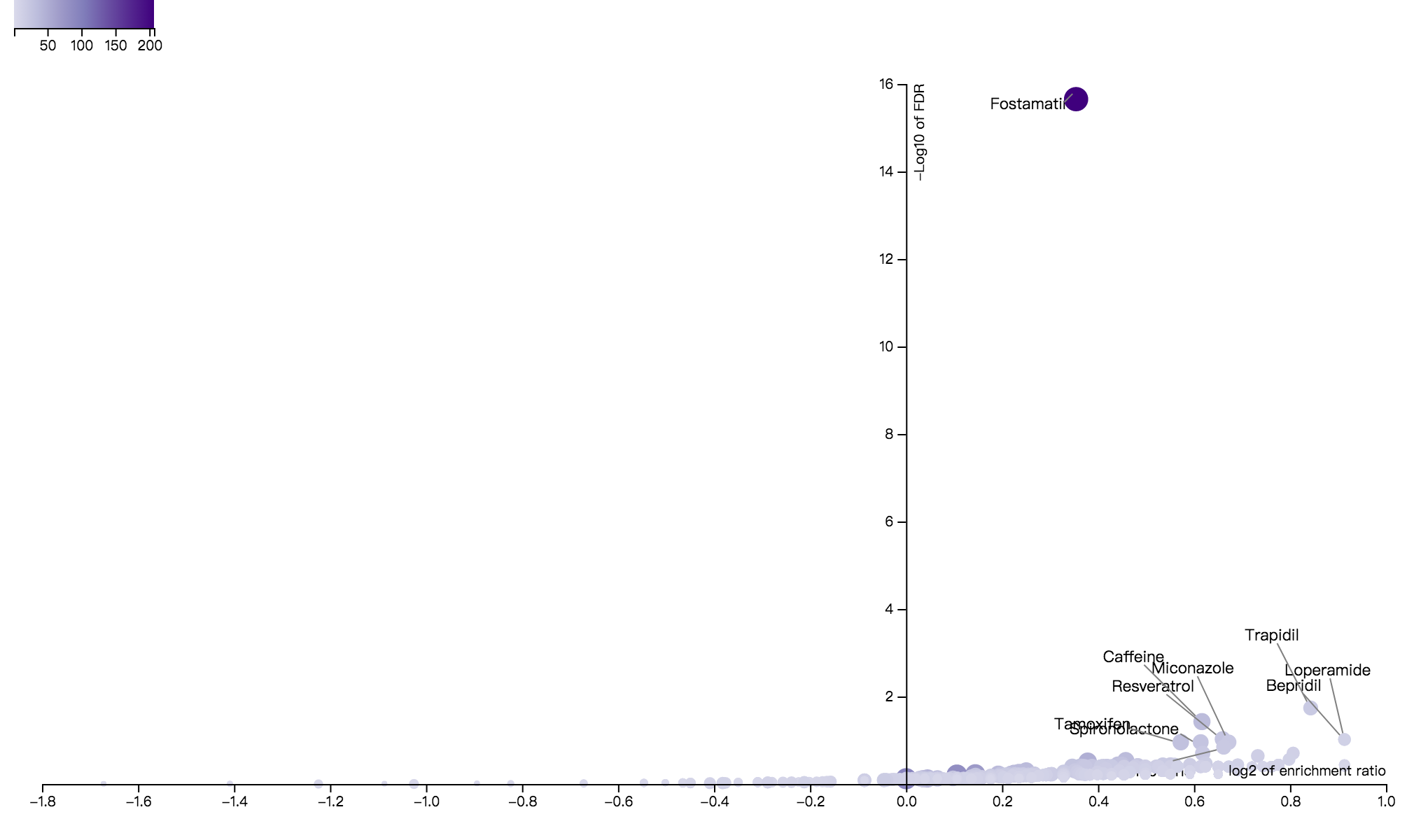


X axis: log2 of enrichment ratio;

Y axis: -log10 of FDR;

Dot shade: overlap value;

Figure captions: 10 terms were significantly marked.

4 Drug Database:GLAD4U

4.1 Bar chart:10 terms were significantly enriched


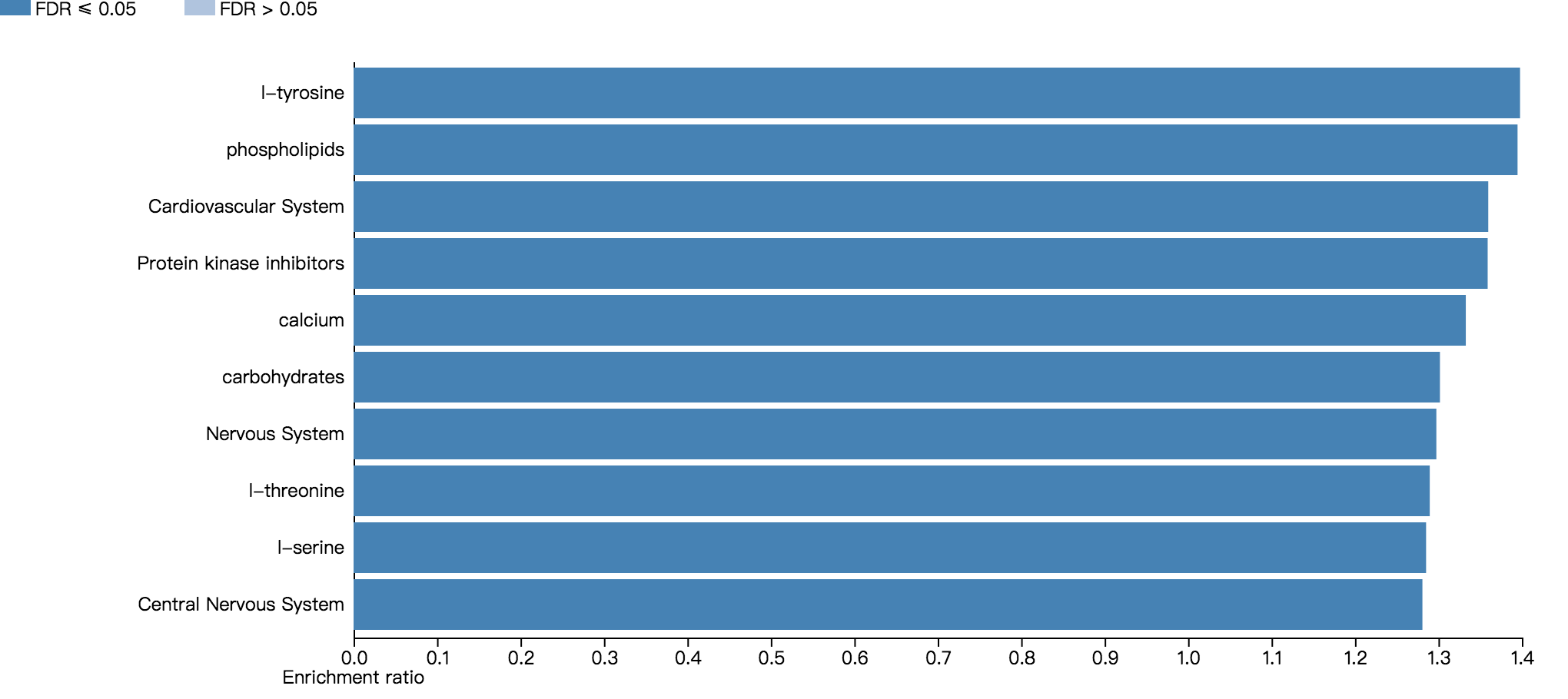


4.2 Volcanoplot:10 terms were significantly marked


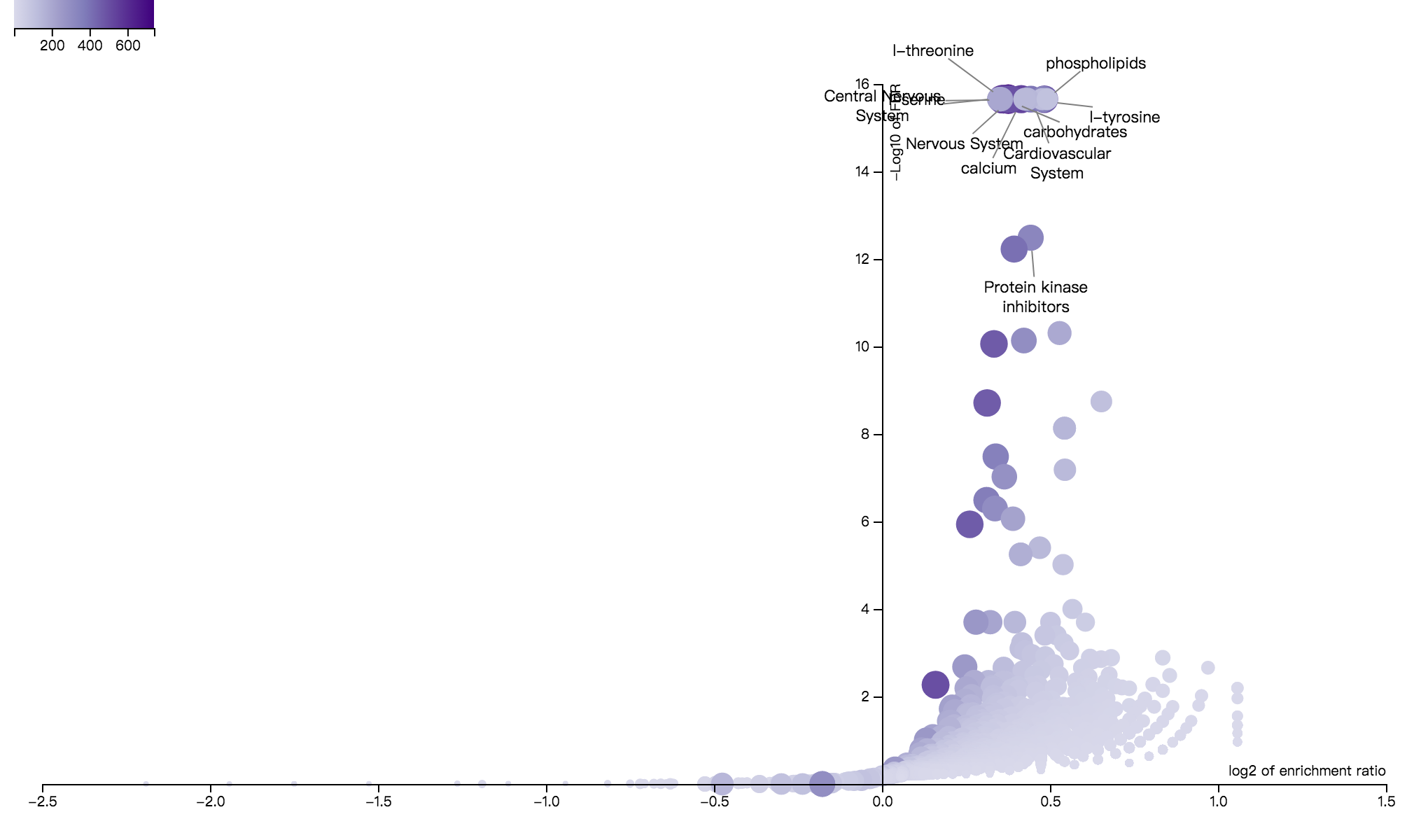


X axis: log2 of enrichment ratio;

Y axis: -log10 of FDR;

Dot shade: overlap value;

Figure captions: 10 terms were significantly marked.
