# Supplementary material for: Methylation of CYP1A1 and VKORC1 promoter associated with stable dosage of warfarin in Chinese patients
Source: PeerJ. 2021 Jun 22;9:e11549. doi: 10.7717/peerj.11549 (PMC8231338; doi:10.7717/peerj.11549)
Supplement: Supplemental Information 4 [file peerj-09-11549-s004.pdf]

Miame Checklist

Part 1 Experiment description

Patients type

Patients who were on stable warfarin treatment from a biobank of Chinese patients treated at the Zhongshan Hospital, School of Medicine, Xiamen University and the Cardiovascular Hospital, School of Medicine, Xiamen University.

experimental variables (runners vs. non-runners, high fat vs. low fat)

The study subjects were assigned to "low-dose" (≤2 mg/day), "medium-dose" (2 mg/day to 4 mg/day), and "high-dose" (≥4 mg/day) groups, on the basis of the stable dose of warfarin refer to the previous study in China.

n-count

473,920 of the 485,577 probes (97.60%) were detected with a detection P-value of 0.05 or less, after the probes on the X and Y chromosome were removed..

tissues used for slide

DNA was extracted from peripheral blood cells.

mouse age, and other variables (wean weight, pooled samples, etc.)

Reported in the article results section:Patient characteristics.

Part 2 Array design.

Array series

Human Methylation 450K BeadChip.

Deconvoluted spot list with gene names

The list of Infinium Human Methylation 450K BeadChip can be downloaded in the website:

https://support.illumina.com.cn/downloads/humanmethylation450_15017482_v1-2_product_files.html?langsel=/cn/

This download contains the manifest files (.csv and .bpm) for Infinium HumanMethylation450 v1.2 BeadChip.

Array type (mouse, human, cDNA, oligo, number of genes)

Human DNA methylation.

Array size

485,577 probes.

Slide type (and coating)

Infinium HD Methylation.

Part 3 Samples

Cy3/Cy5 labels for tissues

This is a two-color assay. The Infinium HumanMethylation450 BeadChip assay includes Infinium I and Infinium II study designs. In the latter case, a single base extension from the 3' end of the

probe sequence (which is one base upstream of the query base) will result in either a red or green signal depending on whether the query site was unmethylated or methylated.

The detailed information of Infinium Human Methylation 450K BeadChip can be reviewed in the website:

https://support.illumina.com.cn/content/dam/illumina-marketing/documents/products/product_information_sheets/product_info_hm450.pdf

Dye swap? Or reference control?

The low dose group and high dose group were defined as case groups,and the medium dose group was defined as control group.

The detailed information of Infinium Human Methylation 450K BeadChip can be reviewed in the website:

https://support.illumina.com.cn/content/dam/illumina-marketing/documents/products/product_information_sheets/product_info_hm450.pdf

Labelling protocol used

1. Fill the water circulator.

2. Select Robot QC Tasks | Circulator Manager to set to 44°C.

3. Select XStain Tasks | XStain HD BeadChip.

4. If imaging the BeadChip immediately after the staining process, turn on the scanner.

5. Add the reagents to reservoirs.

6. Invert the XC1, XC2, TEM, STM, and ATM tubes to mix. Remove the caps, and place on the robot deck.

7. Enter the number of BeadChips.

8. Select Run.

9. [Non-LIMS] Enter the stain temperature listed on the STM tube.

10. Place the flow-through chambers into the chamber rack.

11. Select OK.

12. Remove the flow-through chambers from the chamber rack.

13. Set up two top-loading wash dishes labeled PB1 and XC4.

14. Add 310 ml PB1 to the PB1 wash dish.

15. Submerge the staining rack in the wash dish.

16. Leave the staining rack in the wash dish.

17. Disassemble each flow-through chamber.

18. Place the BeadChips into the submerged staining rack.

19. Slowly lift the staining rack 10 times.

20. Soak for 5 minutes.

21. Vigorously shake the XC4 bottle.

22. Add 310 ml XC4 to the XC4 wash dish and cover.

23. Transfer the staining rack from the PB1 to the XC4.

24. Slowly lift the staining rack 10 times.

25. Soak for 5 minutes.

26. Remove the staining rack and place it onto the tube rack.

27. Dry each BeadChip as follows.

a. Grip the BeadChip by the barcode end.

b. Place onto a tube rack with the barcode facing up and toward you.

28. Place the tube rack into the vacuum desiccator.

29. Dry the BeadChips for 50–55 minutes at

675 mm Hg (0.9 bar).

30. [LIMS] Select Infinium HD Methylation | Coat BC2.

c. Scan the barcodes.

The detailed information of Infinium Human Methylation 450K BeadChip can be reviewed in the website:

https://support.illumina.com.cn/content/dam/illumina-marketing/documents/products/product_information_sheets/product_info_hm450.pdf

Sample extraction protocol used

We isolated DNA from peripheral blood cell samples by using the QIAamp DNA Blood Mini Kit (Cat.#51306; Qiagen, German). DNA bisulfite conversion was done in a bisulfite batch with the Zymo EZ DNA MethylationTM Kit (Cat.#D5001, Zymo, USA). Genome-wide DNA methylation was measured by Illumina Infinium HumanMethylation 450 K BeadChip (Cat.#WG-314-1001, Illumina, USA).

Please refer to the online production manual for specific experimental operation.

Amount of sample labelled

We included 30 patients to measure genome-wide DNA methylation and screen for potential warfarin dose-associated CpGs among the three groups (low-dose: 10, medium-dose: 10; high-dose: 10).

Part 4 Hybridizations

Hybridization protocol

1. Incubate the MSA4 plate at 95 °C on the heat block for 20 minutes.

2. Cool at room temperature for 30 minutes.

3. Pulse centrifuge at 280 g.

4. Place the gasket into the hybridization chamber.

5. Add 400 µl PB2 into each reservoir.

6. Place the hybridization chamber insert into the hybridization chamber.

7. Immediately cover the chamber with the lid.

8. [LIMS] Select Select Infinium HD Methylation | Confirm for Hyb.

9. [LIMS] Scan the barcodes.

10. Remove all BeadChips from packaging.

11. Place BeadChips into the robot BeadChip alignment fixtures.

  Select task name on UI Tasks | Hyb-Multi BC2.

12. Place the robot BeadChip alignment fixtures onto the robot deck.

13. Pulse centrifuge the MSA4 plate at 280g.

14. Place the MSA4 plate onto the robot deck.

15. Select Run.

16. Place each robot tip alignment guide on top of each robot BeadChip alignment fixture.

17. To start the run, select OK.

18. When complete, select OK.

19. Remove the robot BeadChip alignment fixtures.

21. Place each BeadChip in a hybridization chamber insert.

22. Place the lid on the chamber and secure with the metal clamps.

23. [LIMS] Select Infinium HD Methylation | Prepare Hyb Chamber. a. Scan the barcodes.

The detailed information of hybridization of Infinium Human Methylation 450K BeadChip can be reviewed in the website: https://support.illumina.com/content/dam/illumina-support/documents/documentation/chemistry_documentation/infinium_assays/infinium_hd_methylation/infinium-methylation-auto-checklist-1000000077299-04.pdf

ALL modifications and deviations from the protocol

None.

Manual hybridization or automatic chamber?

Manual hybridization.

Number of slides done at the same time

12 samples for one BeadChip.

Hyb time

Incubate the MSA4 plate at 95 °C on the heat block for 20 minutes. Cool at room temperature for 30 minutes.

Number of washes

1. Submerge the wash rack in the PB1 wash.

2. Remove the hybridization insert.

3. Remove the BeadChips.

4. Remove the cover seals from the BeadChips.

5. Place the BeadChips into the submerged wash rack.

6. Move the wash rack up and down for 1 minute.

7. Move the wash rack to the next PB1 Wash.

8. Move the wash rack up and down for 1 minute.

9. Confirm that you are using the correct Infinium glass back plates and spacers.

10. Fill the BeadChip alignment fixture with 150 ml PB1.

11. For each BeadChip, place one black frame into the BeadChip alignment fixture.

12. Place each BeadChip into a black frame.

13. Place a clear spacer onto the top of each BeadChip.

14. Place the alignment bar onto the alignment fixture.

15. Place a clean glass back plate on top of each clear spacer.

16. Secure each flow-through chamber assembly with metal clamps.

17. Remove the assembled flow-through chamber from the alignment fixture.

18. Trim the spacers from each end of the assembly.

19. Leave assembled flow-through chambers on the lab bench.

20. Wash the hybridization chamber reservoirs with DI H2O.

Amount of labelled sample hybridized

From the screening cohort, on the basis of the stable warfarin dose, three groups (low-dose: 10, medium-dose: 10;high-dose: 10) were matched by the patients‘ characteristics. We screened for

potential warfarin dose-associated CpGs by comparison of genome-wide DNA methylation between the case groups(low dose group and high dose group) and control groups(medium dose group).

Labelling efficiency

On the array, 473,920 of the 485,577 probes (97.60%) were detected with a detection P-value of 0.05 or less, after the probes on the X and Y chromosome were removed.

Part 5 Measurements

Which version of scanner software used

HiScanSQ:

Scan Settings:Methylation NXT.scst; iScan Control Software: v3.2.45+; AutoLoader2 / AutoLoader2 Service: v2.3.0 / 2.3.0;

Illumina's Infinium Assay for Methylation: GenomeStudio Methylation Module(version 1.8)

Laser power for scan

The detailed information of scan setting of Infinium Human Methylation 450K BeadChip can be reviewed in the website:

https://support.illumina.com.cn/content/dam/illumina-marketing/documents/products/product_information_sheets/product_info_hm450.pdf.

In addition, Approximate Array Scan Time (saving locally): 60 min per BeadChip.

Instrument model numbers

HiScanSQ System SY-103-2001, HiScan Reader SY-103-1001, and SQ Module SY-101-2001.

Must save original .tiff format images (composite image is optional)

Yes. The related files of DNA methylation data can be reviewed at FigShare, Project Link: https://figshare.com/projects/Methylation_of_CYP1A1_and_VKORC1_promoter_associated_with_stable_dosage_of_warfarin_in_Chinese_patients/94196

Normalization protocol

Normalization algorithms transform sample signals in order to minimize the effects of variation arising from non-biological factors. However, when using the GenomeStudio Methylation Module to analyze your data, it is also possible to use raw data without applying a normalization algorithm.

In gene expression analysis, most normalization algorithms operate under the assumption that the majority of genes are not differentially expressed. However, we cannot make this same assumption for the purposes of methylation analysis. For this reason, the GenomeStudio Methylation Module offers simple normalization algorithms that do not rely on the assumption that the majority of genes are differentially expressed. The following sections of this chapter contain detailed descriptions of the GenomeStudio Methylation Module normalization algorithms used for methylation analysis:

Average , Controls and Background.

Average normalization is applied across Sentrix Array Products, with the goal of minimizing scanner-to-scanner variation. The assumption is that biological conditions are balanced across Sentrix Array Products. Suppose that you are using three SAMs. Let 11, 12, and 13 be the average intensity values for the first color channel of each SAM, and let m equal the average across SAMs. The average is computed across all loci and all bundles, so m = (11+12+13)/3. Multiply the intensity values of color channel 1 in SAM 1 by m/I1. Normalize the other two SAMs and the other color channel in the same way. At the end of this procedure, every SAM has the same average intensity in

each color channel. When the first SAM is divided by I1, the mean is scaled to 1. When it is multiplied by m, the mean is scaled to m. In other words, the three SAMs initially have mean intensity values of I1, I2, and I3, but after scaling, they all have mean m.

Normalization to Internal Controls

This method is available for the Infinium HumanMethylation450 assay. Normalization control probe pairs are designed to target the same region within housekeeping genes and have no underlying CpG sites in the probe. One probe will extend to incorporate a base in Green channel, and the corresponding probe will incorporate a base in the Red channel. Over 90 probe pairs are used for normalization. ` Normalization values are calculated and used separately in two channels separately. For the Green channel, CG controls values are used; for the Red channel, AT controls values are used. ` For normalization, probe intensity in the given sample is multiplied by a constant normalization factor (for all samples) and divided by the average of normalization controls in the probe's channel in the given sample. ` The normalization factor is calculated as the average of AT and CG normalization controls in sample 0, the reference sample. It is the first sample in the list of samples. It does not matter which sample is use, as long as there is one reference sample. ` Outliers are not removed for normalization controls.

Does the scanner software subtract background? How much?

The background value is derived by averaging the signals of built-in negative control bead types. Outliers are removed using the median absolute deviation method. Background normalization is capable of minimizing the amount of variation in background signals between arrays. This is accomplished using the signals of built-in negative controls, which are designed to be thermodynamically equivalent to the regular probes but lack a specific target in the transcriptome.

Negative controls allow for estimating the expected signal level in the absence of hybridization to a specific target. The average signal of the negative controls is subtracted from the probe signals. As a result, the expected signal for unexpressed targets is equal to zero. Half of the unexpressed targets are expected to have negative signals because the average signal of negative controls is subtracted. Assuming symmetry, half the negative controls are lower than average, and half are higher. Therefore, half of the negative controls will be negative after the average is subtracted. The negative controls represent unexpressed targets, half of which are expected to be negative after subtraction. For HumanMethylation450 array, background subtraction is calculated differently. ` Background is calculated in two channels separately. ` Channel background is 5% percentile of the negative controls in the given channel. Negative probes outliers are not removed. ` Background is subtracted from probe intensities in the same channel. If intensity becomes negative, it is set to 0. ` Background is also used in probe detection calculations. In this case, backgrounds are subtracted from negative controls only. This guarantees that probe detection does not change with background subtraction. If the intensity of a negative control becomes negative, it is set to 0. There might be slight changes in detection p-values between data sets with and without background subtraction. This might happen because of the nonlinear nature of replacing negative values of probes and negative values of controls with 0.

Spot raw values, background intensity, ch1 and 2 intensity, etc.

The Infinium HumanMethylation450 BeadChip assay includes Infinium I and Infinium II study designs. In the latter case, a single base extension from the 3' end of the probe sequence (which is one base upstream of the query base) will result in either a red or green signal depending on whether the query site was unmethylated or methylated.

Corresponding gene name

The list of Infinium Human Methylation 450K BeadChip can be downloaded in the website:

Files<https://support.illumina.com.cn/downloads/humanmethylation450_15017482_v1-2_product_files.html?langsel=/cn/>;

This download contains the manifest files (.csv and .bpm) for Infinium HumanMethylation450 v1.2 BeadChip.

Methods of analysis (MAN, Spotfire, Genespring) be detailed.

All differential methylation analysis algorithms compare a group of samples (referred to as the condition group) to a reference group. This comparison is made using the following error models: Illumina Custom Model . This model operates under the assumption that the methylation value ß is normally distributed among replicates corresponding to a set of biological conditions. The variation in the estimate of ß is a function of ß. The function was estimated for all values of ß by repeatedly measuring loci with known methylation fractions ranging from 0 to 1, and then fitting a parabola to the standard deviation as a function of ß. We produce p-values using the following approach: where z is the two-sided tail probability of the standard normal distribution. A diff score for a probe is computed as: For a locus with multiple probes, the DiffScores across probes are averaged. In addition, a concordance value between probes is reported.

Normalized to controls? Controls removed? All normalization parameters

The following sections include descriptions of the Infinium Methylation Controls: Staining Controls; Extension Controls; Hybridization Controls; Target Removal Controls; Bisulfite Conversion Controls; G/T Mismatch Controls (HumanMethylation27); Specificity Controls; Negative Controls; Non-polymorphic Controls.

The detailed information of parameters of Infinium Human Methylation 450K BeadChip can be reviewed in the website:

https://support.illumina.com.cn/content/dam/illumina-marketing/documents/products/product_information_sheets/product_info_hm450.pdf.

Name of Images, Experiment, and location of files.

The related files of DNA methylation data can be reviewed at FigShare, Project Link:

https://figshare.com/projects/Methylation_of_CYP1A1_and_VKORC1_promoter_associated_with_stable_dosage_of_warfarin_in_Chinese_patients/94196

Lowess or other normalization if used (and parameters)

None.

Output file

The output files of DNA methylation data can be reviewed at FigShare, Project Link:

https://figshare.com/projects/Methylation_of_CYP1A1_and_VKORC1_promoter_associated_with_stable_dosage_of_warfarin_in_Chinese_patients/94196

Normalized ratios

Normalization control probe pairs are designed to target the same region within housekeeping genes and have no underlying CpG sites in the probe. One probe will extend to incorporate a base in Green channel, and the corresponding probe will incorporate a base in the Red channel. Over 90 probe pairs are used for normalization. Normalization values are calculated and used separately in two channels separately. For the Green channel, CG controls values are used; for the Red channel, AT controls values are used. For normalization, probe intensity in the given sample is multiplied by a constant normalization factor (for all samples) and divided by the average of normalization controls in the

probe's channel in the given sample. The normalization factor is calculated as the average of AT and CG normalization controls in sample 0, the reference sample. It is the first sample in the list of samples. It does not matter which sample is use, as long as there is one reference sample. Outliers are not removed for normalization controls.

Numerical manipulations

The average β-value was estimated as the proportion of total signal intensity from the methylated-specific probe to represent the methylation level (range 0–1). Delta beta (Δβ) was used to represent the difference in methylation levels among the case groups(low dose group and high dose group) and control group(medium dose group).

Cut off values

To avoid false positives, the probes were filtered out using a detection P-value of less than 0.05.

Significant differences were established with a FDR adjusted DiffScore ≥ ｜13｜~ P ≤ 0.05.

Part 6 Normalization controls

Hypothesis

To investigate the association between DNA methylation and the stable warfarin dose through genome-wide DNA methylation analysis.

Gene expression patterns found

CpGs surrounding the xenobiotic response element (XRE) within CYP1A1 promoter methylation levels significantly differed between the different dose groups ($P < 0.05$), and these CpGs presented a positive correlation ($r > 0$, $P < 0.05$) with an increase in the stable dose of warfarin. At the VKORC1 promoter, two CpGs methylation level were significantly different statistically between

the differential dose groups ($P < 0.05$), and one CpG(Chr16: 31106793) presented a significant negative correlation ($r < 0$, $P < 0.05$) among different dose (low, medium, and high) groups.

Controls used, normalization methods used (see above)

We screened for potential warfarin dose-associated CpGs by comparison of genome-wide DNA methylation between the case groups(low dose group and high dose group) and control groups(medium dose group).
